# Dynamic needs and challenges of people with drug-resistant tuberculosis and HIV in South Africa: a qualitative study

**DOI:** 10.1016/S2214-109X(20)30548-9

**Published:** 2021-04

**Authors:** Amrita Daftary, Shinjini Mondal, Jennifer Zelnick, Gerald Friedland, Boitumelo Seepamore, Resha Boodhram, K Rivet Amico, Nesri Padayatchi, Max R O’Donnell

**Affiliations:** Dahdaleh Institute of Global Health Research, School of Global Health, York University, Toronto, ON, Canada (A Daftary PhD); Centre for the Aids Programme of Research in South Africa MRC-HIV-TB Pathogenesis and Treatment Research Unit, Durban, South Africa (A Daftary, R Boodhram MMedSc, N Padayatchi MD, M R O’Donnell MD); Faculty of Medicine, McGill University, Montreal, QC, Canada (S Mondal MPH); Graduate School of Social Work, Touro College and University System, New York, NY, USA (J Zelnick PhD); Yale School of Medicine, Yale University, New Haven, CT, USA (G Friedland MD); Department of Social Work, University of KwaZulu-Natal, Durban, South Africa (B Seepamore PhD); School of Public Health, University of Michigan, Ann Arbor, MI, USA (KR Amico PhD); Division of Pulmonary, Allergy, and Critical Care Medicine (M R O’Donnell) and Department of Epidemiology, Mailman School of Public Health (M R O’Donnell), Columbia University Irving Medical Center, New York, NY, USA

## Abstract

**Background:**

There is little evidence of patient acceptability for drug-resistant tuberculosis (DRTB) care in the context of new treatment regimens and HIV co-infection. We aim to describe experiences of DRTB-HIV care among patients in KwaZulu-Natal province, South Africa.

**Methods:**

In this qualitative study using Bury’s framework for chronic illness, we conducted 13 focus groups at a tertiary hospital with 55 patients co-infected with DRTB and HIV (28 women, 27 men) who were receiving new bedaquiline-based treatment for DRTB, concurrent with antiretroviral therapy. Eligible patients were consenting adults (aged >18 years) with confirmed DRTB and HIV who were enrolled into the PRAXIS study within 2 weeks of initiating bedaquiline-based treatment for DRTB. Participants were recruited from the PRAXIS cohort to participate in a focus group based on their time in DRTB treatment: early (2–6 weeks after treatment initiation), middle (2–6 months after discharge or treatment initiation if never hospitalised), and late (>6 months after treatment initiation). Focus groups were carried out in isiZulu language, audio recorded, and translated to English within 4 weeks. Participants were asked about their experiences of DRTB and HIV care and treatment, and qualitative data were coded and thematically analysed.

**Findings:**

From March, 2017, to June, 2018, distinctive patient challenges were identified at four critical stages of DRTB care: diagnosis, marked by centralised hospitalisation, renunciation from routine life, systemic stigmatisation and, for patients with longstanding HIV, renewed destabilisation; treatment initiation, marked by side-effects, isolation, and social disconnectedness; discharge, marked by brief respite and resurgent therapeutic and social disruption; and continuity, marked by deepening socioeconomic challenges despite clinical recovery. The periods of diagnosis and discharge into the community were particularly difficult. Treatment information and agency in decision making was a persistent gap. Sources of stigmatisation shifted with movement between the hospital and community. Resilience was built by connecting to peers, self-isolating, financial and material security, and a focus on recovery.

**Interpretation:**

People with DRTB and HIV undergo disruptive, life-altering experiences. The lack of information, agency, and social protections in DRTB care and treatment causes wider-reaching challenges for patients compared with HIV. Decentralised, community, peer-support, and differentiated care models for DRTB might be ameliorative and help to maximise the promise of new regimens.

**Funding:**

US National Institutes of Health.

## Introduction

The diagnostic and treatment complexity, morbidity, and mortality associated with drug-resistant tuberculosis (DRTB) render it the most challenging form of the disease. Each year about half a million people develop multidrug-resistant tuberculosis (MDRTB) that is resistant to the first-line anti-tuberculosis medications isoniazid and rifampicin. 10% of patients are additionally resistant to a fluoroquinolone and second-line injectables, and have extensively drug-resistant tuberculosis (XDRTB).^1^ Treatment success in DRTB is very low (28–52%).^1^ The treatment duration—which until recently spanned 18–24 months—medication adverse effects, isolation, and accompanying financial, mental, and social hardships are major barriers for patients.^2^

In South Africa more than 322 000 new cases of tuberculosis are reported per year, and 4.4% of incident cases are drug resistant. About 60% of patients with tuberculosis also live with HIV.^1^ In 2015, the National Tuberculosis Program began introducing shorter regimens of 9–12 months for MDRTB (18 months for XDRTB), gradually leading to regimens in which second-line injectable agents were replaced with oral medications, notably bedaquiline.^3,4^ The literature regarding patient acceptability of new DRTB regimens is still sparse. The multidrug course remains lengthy, and the therapeutic needs of patients living with HIV are not well documented. Studies characterising challenges in DRTB treatment offer cross-sectional snapshots but seldom examine changes over time that would inform patient-centred care.^2,5–7^ To address these gaps and to guide interventions, we describe longitudinal experiences in DRTB care and treatment among patients receiving new regimens concurrently with antiretroviral treatment.

## Methods

### Study design

This qualitative study drew on Bury’s theory of biographical disruption, developed to examine critical changes and disruptions in the expectations, identities, relationships, plans, and structures of daily life among people living with a chronic debilitating illness; and processes by which patients seek to repair disruptions to regain their social identity and status.^8^ The complexity of DRTB treatment, alongside co-occurrence with HIV, position it to upset the order of patients’ day-to-day lives. Hence, Bury’s framework was apt to chronologise patients’ lived experiences, in tandem with longitudinal methods of phenomenological qualitative inquiry.^9–11^

This study was nested into the observational cohort of the PRAXIS study (Promoting Engagement in the DRTB-HIV Care Continuum in South Africa, NCT03162107), a mixed methods study of adherence assessment in DRTB-HIV treatment at a centralised tertiary hospital in KwaZulu-Natal province. The PRAXIS study was approved by research ethics committees at the University of KwaZulu-Natal (BE242/16) and Columbia University (IRB-AAAQ5753).

### Participants

From November, 2016, to February, 2018, 200 consenting adults (>18 years) with confirmed DRTB and HIV were enrolled into the PRAXIS study within 2 weeks of initiating bedaquiline-based treatment for DRTB, as per national guidelines.

In PRAXIS, patients were hospitalised and discharged after culture conversion at the discretion of site physicians and received antiretroviral treatment as per standard of care. Fully ambulatory treatment was uncommon during this early period of bedaquiline roll-out. During inpatient hospitalisation, patients self-administered their medications but with supervision or support from hospital staff. Upon discharge, they collected treatments from the site every month, and self-administered. Services for DRTB and HIV were run out of separate clinics in the same medical complex. Antiretroviral treatment was switched from a fixed-dose combination to non-fixed-dose combination regimen to avoid drug–drug interactions with bedaquiline.^12^

About 5 months after commencement of the PRAXIS study, a sub-sample of PRAXIS participants were invited by a study facilitator to participate in a focus group based on their time in DRTB treatment: early (2–6 weeks after treatment initiation), middle (2–6 months after discharge or treatment initiation if never hospitalised), and late (>6 months after treatment initiation). Recruitment categories were kept broad at the outset because participants had varied individualised regimens and hospitalisation periods, and we sought to characterise the events or stages in care that they perceived to be most relevant. Recruitment was also guided by the principle of saturation,^13^ expected to be achieved with 12 focus groups.

Focus groups were included in the written informed consent procedure for the observational cohort. Verbal permission was individually sought and obtained from potential focus group participants, with opportunity to ask questions and decline without impacts on their medical care or study status. Focus group participants received refreshments and ZAR150 (US$12); outpatients also received transport reimbursement.

### Procedures

**Figure 1:**
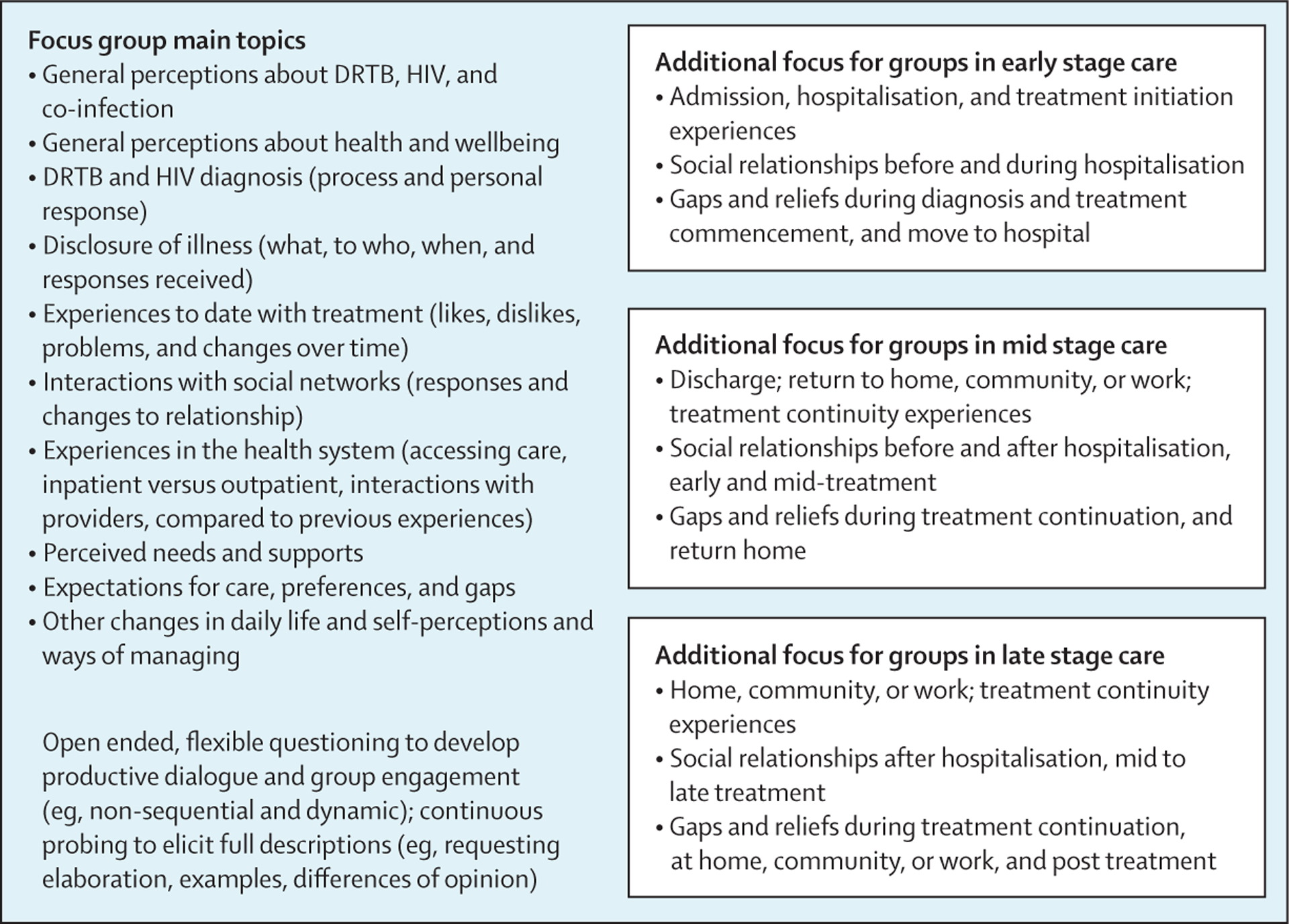
Focus group topic guide DRTB= drug-resistant tuberculosis.

Focus groups^14^ were carried out in isiZulu language in a ventilated private room on-site at the tertiary care hospital, and audio recorded. The facilitator posed questions drawing on distinct focus group guides for each stage (figure 1). A moderator assisted with logistics and note-taking. Both the facilitator and moderator were study staff members extensively trained in qualitative interviewing and group facilitation, and not involved in patient care or adherence. The wording and sequencing of questions and conversation prompts (ie, probes) were adjusted to suit evolving group dynamics. Participants were encouraged to share examples and contrasting perspectives.^13,14^ Small groups of 4 to 8 participants were planned to enable infection control, and together with pseudonyms and ice breakers, group rapport and trust. It was expected that some participants would be unavailable for sessions, after agreeing to participate.

Recordings and notes were transcribed verbatim by data collectors, deidentified, cross-checked for accuracy within a week of each focus group, and translated to English within 4 weeks with attention to metaphors and linguistic subtleties. Preliminary analysis was iterative, concurrent with data collection, to refine probing and assess saturation (saturation was achieved in the 11th focus group) via debriefing with data collectors, and review of focus group notes, transcripts, and recordings.^13,15^ Data were then entered into NVivo 12 (QSR International) for comprehensive thematic analysis.^5,16,17^ A coding scheme was derived from focus group topics, leaving space for inductive analysis; memoing enabled higher-level comparisons. Codes were categorised under broader concepts, using discursive techniques to identify and question patterns, assess context, and achieve conceptual clarity.^15,16^ Concepts were juxtaposed with aspects of care found to be relevant by participants. Bury’s framework^8,11^ informed further thematic articulation and refinement.

Preliminary analysis was done by AD and BS. Coding was implemented independently by AD and SM, and then together to enhance reliability. JZ and GF were successively involved in concept and theme development. BS continuously investigated linguistic and lexical nuances in consultation with data collectors because they were fluent in English and isiZulu and from the study setting. Emerging ideas were interrogated through reflexive practice and systematic consultations with the full team to further strengthen analytic dependability, confirmability, and trustworthiness.^13,15,17^ Reporting adheres to Consolidated Criteria for Reporting Qualitative Research guidelines.^18^

### Role of the funding source

The funder of the study had no role in the study design, data collection, data analysis, data interpretation, or writing of the report.

## Results

From March, 2017, to June, 2018, 13 focus groups (four early-stage care, three middle-stage care, six late-stage care), each with 3 to 7 participants of the same sex, were completed with 55 unique patients with DRTB and HIV (30 females in seven focus groups, 25 males in six focus groups). Sessions averaged 110 min (range 65–135). Patients were all diagnosed with HIV and receiving antiretroviral treatment before DRTB diagnosis and bedaquiline initiation (table 1). All patients who were approached agreed to participate, although 1 to 2 patients per focus group did not attend because of another appointment or feeling unwell. Four patients joined a second focus group when they progressed into a different stage of care and their responses were not dissimilar from other co-participants.

**Figure 2:**
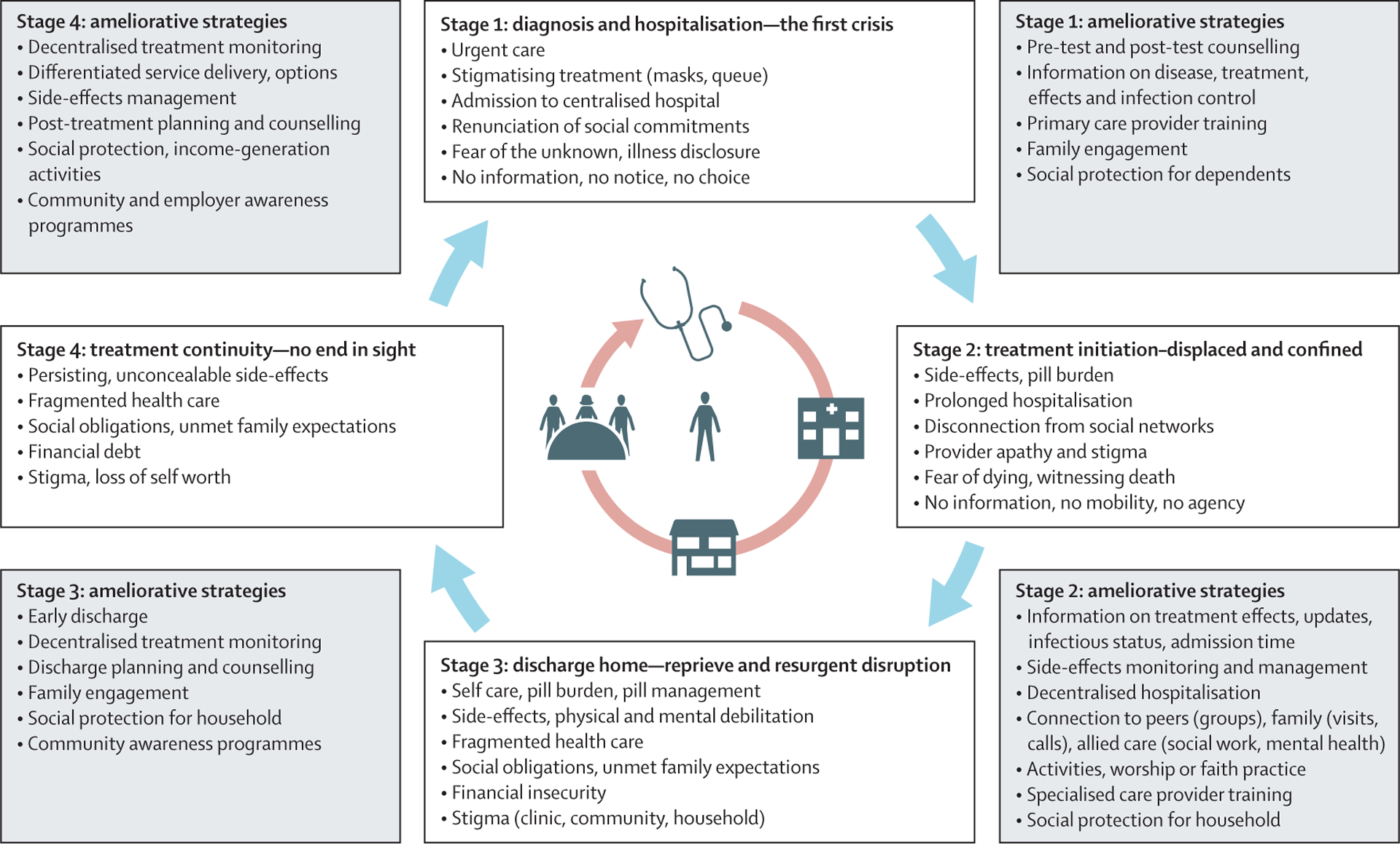
Chronology of disruptions during DRTB treatment in people with DRTB and HIV, and recommendations for amelioration DRTB=drug-resistant tuberculosis.

HIV and DRTB severely altered the day-to-day lives, or biographies, of participants, affecting their identities, bodies (physical and mental health), social relationships, and finances, with temporal shifts over time (stage in care) and location (hospital or home). Participants were recruited based on a broad interpretation of their time on DRTB treatment. However, participants’ narratives led us to organise critical situations or events that they perceived to be disruptive and ameliorative into four stages that overlap with the typical chronology of DRTB illness, beginning at the point of diagnosis (figure 2). Differences tied to participants’ gender or infection are noted where emergent. Select representative quotes and their conceptual relevance are distinctly tabulated (table 2).

### Stage 1: diagnosis and hospitalisation—the first crisis

In the first stage, DRTB diagnosis entailed multiple tests at multiple facilities, often with delay. The news was eventually shared with patients through serious, urgent tones, and most patients experienced dual shock when simultaneously informed they would be admitted indefinitely to a central, specialised hospital. Patients were very ill during this time, and diagnosis generally resulted in rapid medical attention. However, the conspicuousness and fear with which patients were handled left them feeling marked and stigmatised. The patients had to wear a face mask, wait in separate queues, stand outside clinics, or move into separate wards. Patients were given little explanation and no choice about hospitalisation, leaving them confused and destabilised. It was not surprising that some refused to start (in the words of one patient, “ducked”) treatment to sort out their life responsibilities.

Because diagnosis of DRTB was a lengthy process, patients confided in at least one person, usually a trusted household relative, to assist with family care or transport. Patients shared few details about tuberculosis drug-resistance. The little information that the patients did have about DRTB—that DRTB was contagious and possibly deadly—incited excess fear. Several patients noted an immediate distance from others after sharing that they had, in their words “big TB”, the colloquial reference for DRTB, which discouraged them from disclosing further, and patients went on to feign excuses for leaving work or their community. Government vehicles and masked nurses, however, visited some patients’ homes, leaving them exposed to public scrutiny. Tuberculosis commonly signalled HIV, compounding their stigmatisation and family shame.

Patients diagnosed with HIV while being investigated for tuberculosis found it easier to disclose tuberculosis, despite the limited information, while they processed their HIV status; HIV was understood to be permanent and more daunting. By contrast, patients who had been living with HIV for a longer period found it difficult to discuss their new diagnosis. These patients had already disclosed HIV to their networks, at times years earlier, overcome changes to their social (primarily, sexual) relationships, adapted to antiretroviral treatment, and developed resilience against threats to their health and identity. The new symptoms and demands of DRTB treatment, however, threatened to disrupt this equilibrium. Disclosing and accepting DRTB was further challenging, regardless of time since HIV diagnosis, because a DRTB diagnosis was accompanied with a relative absence of information compared with that received for HIV. Patients without a history of tuberculosis (who could not rationalise developing a disease linked to treatment non-adherence), those who felt hopeless about future treatments (because they had experienced tuberculosis or tuberculosis treatment failure in the past), and those who felt ostracised or lacked support for their dependants (particularly some mothers), also struggled to accept a diagnosis of DRTB and concurrent mandate to be hospitalised.

Several situations alleviated this early sense of crisis and loss of control. First, was information and counselling, which had softened news about HIV and abated many patients’ fears. Patients wanted similar explanations about DRTB, particularly in their first language, isiZulu. Second, patients did not want to be singled out (eg, separate queues). Being among specialised providers who regarded their diagnosis as routine, or with other patients wearing masks or receiving DRTB treatment, helped to ameliorate their early anxieties.

### Stage 2: treatment initiation—displaced and confined

In the second stage, patients reported feeling comfortable and stable on antiretroviral treatment when commencing DRTB treatment. They complained about having to take more pills more frequently with a new non-fixed-dose combination antiretroviral treatment regimen, from one pill daily to two pills twice daily. However, this compared little with the pills the patients had to take for DRTB, which brought their daily pill count to 22–30. Over and above this pill burden, patients perceived side-effects to be the greatest challenge during the first 3 months of DRTB treatment and, despite concurrent changes to antiretroviral treatment, attributed the side-effects all to DRTB medications. Nausea, stomach upset, itchiness, headache, fatigue, and a faster heartbeat were common complaints, although vomiting and joint or leg pain were felt to be the most disconcerting. Many patients complained of feeling mentally unwell and as if they were on illicit drugs. Changes to vision and skin complexion were less frequent but considered highly undesirable. About a quarter of patients received second-line injectable agents for DRTB (table 1) before initiating bedaquiline and appreciated switching to an all-oral regimen, although one patient insisted the injection was more powerful. These patients still reported side-effects from other medications and a high pill burden, with many experiencing long-term effects from discontinued drugs (eg, hearing loss). Regardless of this history, side-effects prompted several patients to consider treatment discontinuation and a few patients admitted to having developed techniques to feign pill ingestion even while being observed by a nurse.

Disconnection from routine life and relationships was another major source of concern. Aside from few phone calls and text messages, almost all patients were socially cut off. Several patients later appreciated being admitted while acutely unwell, in order to adjust to new treatment schedules. However, extended stays were incomprehensible. Patients spent days being idle, contemplating death or witnessing the death of others with few distractions, choices, or updates about their own progress. Many families lived far away, and relatives appeared disinclined to visit infectious patients. Patients could not help but feel abandoned, with some describing themselves as orphans or prisoners. Mothers, separated from their children, felt especially distraught. Financial concerns rose for patients who had been the main wage earner. A handful enjoyed employer benefits or social grants, which were used up for household expenses, but most patients received no aid.

Patients received counselling about bedaquiline, but most other information about treatment was learned through experience. Patients who had little understanding about their recovery, treatment risks, changes to medications, and discharge, struggled and they attributed this to poor or inconsistent provider communication. Patients feared that providers looked down on them or blamed them for their disease or diseases, or were overworked and dispassionate. Requests for additional information or support (eg, social work) led to patients being dubbed as irritating or informers seeking to lodge a complaint. This contrasted with the attention received for antiretroviral treatment, by dedicated HIV staff.

The emotional and physical upheaval of this stage was alleviated through positive provider and peer interactions where information, advice, empathy, and greetings were exchanged, which convinced many patients to stay on course with their treatments. Sharing personal struggles with peers honed camaraderie and relieved solitude. Prayer, belief in God, and the need to protect one’s family from DRTB were important motivators for patients who described this to be the bleakest moment of their life. Ultimately, patients understood that treatment was their only chance for recovery and came to accept their situation.

### Stage 3: discharged home—reprieve and resurgent disruption

In the third stage, 3–6 months into DRTB treatment, patients felt their bodies adapt. Patients were still weak and side-effects were evident, but they were less frequent and severe than in the earlier stages of treatment. Most patients were discharged (table 1), and celebrated family reunification. However, many experienced difficulties in adjusting to new household routines. Within weeks, the initial enthusiasm of relatives was replaced by resentment, impatience, and disbelief about patients’ persistent debilitated state and inability to contribute to chores while consuming scarce resources. The resurgent damage to patients’ concept of self and relationships jarred with many who believed that the worst of the DRTB treatment had passed.

Patients’ stigmatisation grew by way of avoidance, disrespect, and diminishing support from their family and social networks. Explicit disclosure of patients’ health status to their family and social networks was uncommon, but it was difficult for patients to hide their long absence or changed physical appearance. Neighbours learned they had a serious type of tuberculosis, and it was assumed that anyone with tuberculosis had HIV. Although it was unclear whether patients were devalued because of having one or both illnesses, there was distinct tension about tuberculosis risk—eg, several fathers who had previously disclosed their HIV status were only now denied the opportunity to see their children. Patients thus regularly self-isolated to recuperate and prevent transmission, but also to preserve self-respect and deflect stigma. Feeling confined within one’s own community was more isolating than any experience encountered in the hospital, and some patients moved to live with others who provided more empathy or material assistance. Younger patients had their social lives disappear; they found it tiring to socialise and understood that some activities (eg, drinking alcohol) could compromise recovery. Romantic relationships that petered off while hospitalised officially ended for several patients once they returned home, although many relationships had ended when HIV was diagnosed. A few men suffered losses of libido. Being shunned by girlfriends or disrespected, especially by younger women, threatened their manhood amid financial insecurity.

Several patients reminisced about the advantages of inpatient life where they had connected to other patients, received timely attention including meals and medications, and occasional counselling. Household members were less sympathetic than peers in the hospital, meals were now seldom prepared regularly, and patients were rarely reminded to take their pills. Community social workers were unfamiliar with DRTB. Primary care clinics referred patients back for specialised care as soon as their DRTB status was identified, causing patients to endure tedious ambulance rides and overnight hospital stays for minor intermittent issues (eg, headache). Patients were in the continuation phase of DRTB treatment that involved fewer medications; however, the pill burden was still high and weighed upon them in the wake of persistent fatigue, renewed loneliness, and loss of any adaptation achieved in the hospital. With no one to monitor their progress, several patients missed taking their medications on time or altogether.

Material and emotional support in the home helped to alleviate patients’ difficulties. When relatives empathised and engaged in patients’ upkeep (eg, provided reminders and essential life supplies), patients felt motivated to take active steps towards recovery (eg, eat healthier, avoid alcohol, take pills on time). Some patients made explicit requests for the government to run campaigns about DRTB, just as they did for AIDS, to publicly advocate for their needs.

### Stage 4: treatment continuity—no end in sight

In the fourth stage, which was 8–12 months into treatment, many patients felt healthier. Appetite and weight normalised and pill-taking became integrated into daily routines; however, some side-effects continued to be disruptive. Conspicuous skin pigmentation discouraged some patients from leaving home. Limb pain, difficulty concentrating, difficulty hearing, and mental debilitation prevented some patients from performing basic chores and regaining independence. Side-effects that were tolerated when faced with imminent death or in the company of other patients were now increasingly unacceptable. Prolonged debilitation also reminded patients they were perceivably ill and infectious, with reason for others to maintain a distance.

Deepening financial deprivation was the other major source of stress. Limited grants and benefits had long lapsed. Several patients felt ready to work but opportunities were rare and a history of DRTB was expected to deter employers. Some men found odd jobs (eg, driving taxis) or resorted to stealing to redeem income, and thereby respect, but this interfered with adherence to treatment schedules and clinic appointments. Patients’ dependence on others grew, heightening their self-worthlessness and vulnerability to insidious devaluation, such as loss of voice in household decisions and disparaging conversations about their health.

The later part of DRTB treatment thus continued to challenge many patients who could not yet envision a future in which they would be healthy and productive. Respite came with financial security (eg, relatives’ generosity or work) and recovery from side-effects. A desire to access treatment in convenient and destigmatising ways was frequently voiced. Patients longed to receive care in their community, but equally appreciated the privacy of faraway facilities where they could escape judgment from neighbours and local health-care workers. Patients who felt well voiced hope for a system in which they could receive DRTB treatment akin to other chronic conditions, including HIV, from a local clinic or pharmacy without always needing in-person specialised care. Some patients made the cognitive decision to embrace treatment as a lifeline, necessary for restitution despite stigma and scarce supports. Women were motivated by their children or other ambitions. Men voiced their motivation around ignoring others’ opinions and focusing on their personal interests.

## Discussion

This study chronologises patients’ difficulties and coping actions over the course of DRTB treatment, substantiating a broader body of patient-centred tuberculosis research.^2,5–7,17^ Findings are uniquely rooted in the context of new, all-oral regimens for DRTB, and point to the limits of technical advancements in allaying patients’ challenges. A theoretical framework of chronic illness led us to discern the disruptive potential of critical events along the DRTB care cascade and recommend responsive interventions based on situations that people with DRTB found to be life-altering and ameliorative (figure 2). Women, men, parents, and people with previous tuberculosis or longer-lasting side-effects faced distinct difficulties, some that have been previously documented.^2,19,20^ Patients who had lived with HIV long before developing DRTB and had adjusted their lives to one chronic illness experienced renewed disruption. Biographical reinforcement has been documented in people sequentially diagnosed with multiple chronic conditions.^11^ Here, we saw challenges created by DRTB treatment overwhelm patients who had HIV, even among patients who had a relatively shorter lived experience with HIV, because of the harsher symptoms, treatment (eg, pill burden, side-effects), treatment requirements (eg, hospitalisation, isolation), and poorer access to DRTB-specific information, counselling, and provider attention. These findings underscore differential structures of HIV and tuberculosis health-care programming, and the need to integrate methods of patient, family, and community engagement in settings with a high tuberculosis and HIV comorbidity.^7,21^

The emergence of challenges and patients’ consequent reactions and perceptions towards them were markedly shaped by patients’ position in the DRTB care cascade and location in which care was received. This insight can guide patient-centred interventions. Information and communication gaps were evident throughout DRTB treatment and the effects of this were especially felt during the period of diagnosis and discharge from hospital. A lack of patient agency and self-worth was acute during the period of hospitalisation, and in the latter half of treatment when independence and return to normalcy was increasingly expected. Continuous DRTB treatment counselling and patient engagement could relieve some of these gaps and promote retention in care.^22^ Peer-support programmes might ease transitions from hospital to community,^23^ and extended social protections and return-to-work programmes could alleviate patients’ dependence on others.^24^

Manifestations of stigma also shifted. In the home and community, patients were increasingly disrespected, ignored, and unassisted, whereas in the health system, they were exposed to infection control artifacts that outed them as different if not dangerous; mistrust; and poor provider communication. These experiences have been previously documented.^17,25^ Tuberculosis was routinely linked to HIV, which likely compounded patients’ stigmatisation.^26,27^ We found stigma to deepen during, and despite, recovery while material and social capital exerted a protective effect, corroborating links between stigma, power, and inequity.^28^ Faith-based interventions, that have improved quality of life among patients living with HIV,^29^ and financial aid, could strengthen patients’ resilience to stigma. The COVID-19 pandemic might provide further impetus to uphold family-centred, destigmatising, and rights-based approaches to infectious disease care.^30^

Decentralising DRTB treatment to facilities closer to patients’ homes could ultimately settle many reported difficulties, and all-oral regimens facilitate the expansion of previously implemented community-based and home-based approaches.^3,31–33^ To succeed, a resourced and supportive environment is needed in patients’ homes (dedicated living space, food security, adherence aids, and an informed, empathetic household) and within patients’ community (providers with strong clinical and communication skills, opportunities for income generation, and awareness about DRTB recovery and not just risk). Making space for some patient choice and differentiated care, commonly promoted within HIV programmes and recently postulated for tuberculosis,^34^ could meet the needs of patients who are clinically stable, resourced, and adapted to treatment, diverting attention to those with greater needs and challenges.

Our study has several limitations. We were unable to follow participants over time, and we chose focus groups over private interviews. Consequently, we did not capture individual-level longitudinal data. However, focus groups are increasingly recognised as more effective in uncovering personal disclosures about sensitive topics and were especially suited to the cultural norms of our population,^14,17,35^ demonstrated through participants’ admission about traumatic events and relatively unacceptable social behaviours (eg, poor adherence to treatment). Challenges related to extended hospitalisation or HIV and antiretroviral treatment might be less relevant in ambulatory care or low HIV prevalence settings, although insights into institutional, peer, and HIV-based supports offer opportunities to build patient-centredness into tuberculosis programmes more generally. We did not capture experiences accessing bedaquiline, ending DRTB treatment, or returning to HIV care, which might reveal new or persistent challenges.^5^ The wide data collection period and group-based inquiry also did not support triangulation with adherence data beyond admissions of compromised pill-taking that were shared in some focus groups. Rather, the study provides a rich basis from which patients’ transitions through DRTB and HIV treatment might be better addressed.

The study has informed site provider training^36^ and a multimodal adherence and stigma reduction intervention. This study confirms the need for holistic and reliable institutional, community, and household supports, including drawing on lessons learned in HIV, to meet patients’ longitudinal needs and help maximise the promise of new DRTB drugs.

## Supplementary Material

1

## Figures and Tables

**Table 1:** Participant characteristics

	Total participants (N=55)
Sex	
Female	30 (55%)
Male	25 (45%)
Age, years*	35 (20–62)
Type of tuberculosis	
Multidrug resistant	35 (64%)
Pre-extensively drug resistant	11 (20%)
Extensively drug resistant	9 (16%)
Previous tuberculosis disease	16 (29%)
Previous second-line injectable	14 (25%)
Time on treatment*	
Antiretroviral treatment	23 months (27 days to 10 years)
DRTB†	4 months (17 days to 14 months)
Time left on DRTB treatment*	10 months (13 days to 22 months)
Admission status	
Inpatient	23 (42%)
Outpatient	32 (58%)
Admission history‡	
Ambulatory only	4 (7%)
Hospitalised and ambulatory	51 (93%)
Time hospitalised, days§	86 (16–291)

Data are n (%) and median (range). DRTB=drug-resistant tuberculosis.

* At time of focus group participation.

† Current treatment with bedaquiline-based regimen for DRTB.

‡ Over the full course of DRTB treatment.

§ n=51.

**Table 2:** Conceptual categories of disruption (+) and amelioration (−) and representative quotes reflecting participants’ experiences and perceptions during DRTB-HIV treatment

	Representative quotes
**Stage 1: diagnosis and hospitalisation—the first crisis**	
(−) Marked as different; (−) stigma (health system)	“They treat patients with MDR differently, the moment they heard that I have MDR they started to put their masks on and seated me separately from the others.” FG10_F
(−) Treatment failure (tuberculosis); (−) loss of hope	“I went crazy. I didn’t know whether I should stop taking [current treatment] altogether or hang myself…I thought to myself that after all this time of struggling, all that was for nothing.” FG11_F
(−) Separation from family; (−) no choice or recourse	“She told me that I was to no longer going back home because what I’ve got is contagious, and I said, does this mean I will leave without saying goodbye to my family? And she said I must choose death or this.” FG10_F
(−) Incrimination; (−) abdicated responsibilities	“I was ducking from clinic staff…When they came the final time and found me they came with police officers…thought I was running away…[but] I was rushing…make sure that I have something to keep [my family] going before I leave.” FG1_F
(−) Stigma (home)	“I got discriminated from my family. The feeling of not being loved by your family…as if you are an animal…They did not want me to sit with them… spend time with them or eat with them.” FG1_F
(−) Stigma (community)	“There was just this sudden silence and no one even came to my house…The first people who were supposed to support me left me and even now I am still alone.” FG6_F
(−) Presumption of HIV	“If you tell them that you have MDR they come up with the conclusion that you also have AIDS.” FG1_F
(−) No information (DRTB); (+) information (HIV); (+) information (language)	“All this time I was feeling overwhelmed not knowing what MDR is…They should explain that these are the steps 1, 2, 3 and 4 that you should expect… If they can offer us counselling like with HIV, I will be able to better understand what I’ve got, explain it better to my family and be encouraged to take my treatment.” FG10_F; “It will be better to have a translator who understands our language, so that we can voice out our issues better.” FG3_M
(+) Compassionate care; (+) connection (nurses, peers); (+) normalised like others	“When I finally came here I found that even the nurses were nicer…We like it when the nurses laugh with us because sometimes we think they are isolating us. You end up not being comfortable in that place and that is the most painful feeling, especially when you see that people do not want to be associated with you…I stayed two days here and thought, no, this is coming together nicely.” FG1_F
**Stage 2: treatment initiation—displaced and confined**	
(−) New medicines; (−) new side-effects (physical or mental); (−) pill burden (DRTB, HIV)	“[My] complexion changed to pitch black…[I] threw up a lot…and I had an excruciating headache while taking MDR treatment. The first few months, they gave me the most difficult time…I had to sleep the whole day…I was also losing weight dramatically. There was bedaquiline, it was killing my joints…I think I must have lost my mind when the doctor told me that they had to change my ARV…they added five more [to 18 pills for DRTB], now how many is that?” FG11_F
(−) Separation from child; (−) loss of income; (−) abdicated responsibilities	“It is shocking to hear that you can’t kiss your child anymore especially when you miss them, and what will the child say when her mother cannot kiss her?” FG1_F; “I used to be a driver but lost my job…The owner hired someone else when I got sick so being here for a long time will result in my children suffering because I provide for them.” FG3_M
(−) Disconnection from family; (−) fear of death	“You feel useless since you cannot even see your family… Seeing people die in front of you scares you but if you were at home you were not going to see this. You were going to focus on taking your medication and get well.” FG1_F
(+) Information (HIV); (−) no information (DRTB)	“In the HIV department we are told about our viral load and CD4 count…Being told about your progress really motivates a person…but I did not know anything about my culture results, my MDR results and my sputum results.” FG2_F; “[Providers] said we must not question them…’You know nothing’. They said its part of TB medication. ‘Ask no questions’.” FG5_M
(−) no encouragement or hope	“You don’t even get one person to counsel you and motivate you on what is it that you need to be better in order not to find yourselves in the same situation and also to give you hope.” FG1_F
(−) Stigma (health system)	“When you are severely sick, [providers] gossip about you saying that you have defaulted treatment. Being called a defaulter really hurts because they see you taking medication every day.” FG8_F
(−) Prolonged admission; (+) compassionate care; (+) encouragement; (+) connection (nurses, peers)	“They tell you that you must not go outside the ward, and you really feel like a prisoner.” FG2_F; “I was here for about four months…that broke my heart but there was a nursing sister who treated me like I was her child…She was the only person who took notice of me and she would give me hope and tell me that this too will pass.” FG6_F; “Talking to other people helps you feel better, especially talking to people who are in the same situation as you.” FG1_F
(+) Prayer, faith	“Whenever I would take [medicines], I use to pray first and I found that helped me. I would say, ‘God, you know why you have put me in this situation and you know how you will pull me out of it’.” FG11_F
(+) Protection of family	“I am here because I love my family so much, I didn’t want them to end up getting sick and have this disease…I just want this disease to end with me just like it started with me.” FG4_F
(+) Hospital support; (+) adherence training	“Being admitted in the hospital helped me a lot, I now know that at 8 in the morning I have to take my medication.” FG5_M; “Your heart is telling you that you must be at home, but deep down you knew that you could not abide by the rules on your own.” FG2_F
**Stage 3: discharge home—reprieve and resurgent disruption**	
(+) Abating side-effects; (−) persisting side-effects; (−) pill burden (DRTB)	“In the beginning of treatment there are some side effects that you experience. As you continue, they subside…Even now I do experience some, but they are not as severe.” FG2_F; “I was used to the nurse giving me medication, at home it’s was like my medication are too many! I began to sulk and get angry wherever I took my medication…It felt like the side effects I used to experience came back like the nausea… especially the MDR medication because they are too many.” FG2_F
(−) Slow recovery; (−) prolonged fatigue; (−) loss of productivity; (−) family resentment; (−) loss of family support	“At first my mother showed enthusiasm in helping me take my pills but she is working and can’t be there for me…It is hard to be consistent…side effects also taking its toll…I can’t just be seen as idle so now it is difficult because I am not resting enough.” FG10_F; [“People] have a belief that once you come back from the hospital you must be completely cured. They forget that treating this disease is a process…They treat you well at home for the first months after being discharged but after a couple of months things change. They will say things such as ‘you eat all the time, you are lazy’, forgetting that this disease affects your feet making them sore and it takes time to regain your strength.” FG5_M
(−) Loss of social life; (−) loss of friendship	“It is sad when my friends call me to say let us go to this place and I have to tell them that I can’t come, I have to stay at home. I think this the most hurtful thing about this illness.” FG11_F
(−) Loss of respect, worth; (−) loss of masculinity; (−) stigma (home, community)	“I have a niece who always leaves a room whenever I am there…I just can’t tolerate being disrespected by a girl-child…she knows that I don’t have money. This behaviour makes me feel less of a man.” FG5_M; “When my neighbour’s children come around, they get to be called back, ‘You know that the woman from that house is sick!’ All that doesn’t sit well with me.” FG10_F
(−) Loss of hospital support	“The support that you get from the hospital is not the same with the one you get from home. At the hospital, food comes on time. If it is at 8 then you get it at 8. Whereas at home they can still be cooking by 8…and by that time you haven’t eaten since the morning.” FG7_M “The thing is when you are still in the hospital you desperately miss your family, you miss your life at home…but then again you also remember that you will be treated badly when you come back.” FG5_M
(+) Connection to family; (+) family support; (+) adherence support	“It is very nice to be back home…My uncles buy enough food for us and with a bit of grant that I get I buy myself some nice things that I enjoy eating… My tablets sit on the coffee table. Even my uncles will joke about them and say, ‘These are your sweets my niece, we wish we could take them too’ .” FG6_F
(−) Access to health care; (−) clinic commute; (−) marked as different	“I normally go to our local clinic… for maybe a flu or sore feet…They will treat you differently…tell me that they don’t know about this disease. Yes, but I am not here to get a cure for this disease! …You end up arguing and leave the clinic without getting any form of assistance.” FG7_M; “Getting here [tertiary site] requires me to spend the night at the local hospital then be transported by the ambulance in the morning to pick up my medication…I spend 2 days to and from. We sleep on the benches…one will end up sleeping on the floor.” FG3_F
(−) No information; (+) discharge counselling	“When a doctor knows that you are about to be discharged…[have] the nurses or staff here teach us about our medication…they can make you practise… so that you don’t experience problems when you go back home…because a lot of people are confused.” FG12_M
(+) Information (for patient, family, community)	“We need to have more campaigns like they did with AIDS, have people going from place to place educating people about this disease…and it should be people who have had TB before so that we can share from experience, be straight with people.” FG7_M
**Stage 4: treatment continuity—no end in sight**	
(+) Normalised adherence	“Taking my medication is normal for me now, it’s in my blood. It’s like taking a bath, no one has to tell you that. I know what I have to do.” FG5_M
(−) Persisting side-effects; (−) visible side-effects	“This difficulty seeing thing still troubles me…I can’t read something that’s written down. Then it is my feet. It feels like there is a knot behind them preventing me from walking freely and fast.” FG9_M; “When I meet people they ask, ‘What’s wrong with your eyes. They are red. Are you drunk?… You need to go back to the hospital. You should not be with us here outside.’ …The mother of my children said so long as I am still on this pill, I should find myself another place.” FG9_M; “Everyone asks me and says, ‘What happened to you? Why have you become so dark in complexion?’ And the most annoying part is to explain to them that I am taking pills.” FG6_F
(−) Loss of social grant; (−) dependence on others	“I am struggling financially and sometimes I wish I could have my job back. Doctor utterly refuse to pass grants for us, pills make me eat a lot and I am becoming a burden to those that have income at home.” FG6_F; “You end up being a burden … it’s hard to borrow money from people when you are not working. You end up making many debts to cover other debt.” FG5_M
(+) Employment; (+) mobility; (−) interrupted adherence	“The situation can improve if we are offered temporary employment so that we can contribute at home…If you are lucky to get a disability grant you find that its only valid for six months whereas the treatment is for two years.” FG5_M; “I started working in [new town] so I was not familiar with their clinics… hectic schedule, sometimes my work shift ended at 12 midnight so I did not find the chance to fetch my medication.”FG5_M
(−) Access to care; (−) stigma (health system)	“It would be better to have access to our pills like with patients who are getting ARVs from the shops and local clinics…you can even ask a child to go and collect them for you.” FG7_M; “To collect [treatment] here is better…If it was a queue for those with MDR at a local area clinic, you may stand a great chance to be discriminated against.” FG10_F
(−)(+) Fear of death; (+) focus on recovery; (+) rejection of stigma	“It is the most annoying thing especially taking the TB ones, we force ourselves into taking them…If I had not seen a person who defaulted treatment dying in front of me, I would have defaulted too…but I have embraced them and told myself that this is my life, I have to take care of it.” FG6_F; “What matters for me is being adherent to my medication and not mind other people’s opinions…You must just accept that you are sick and sticking to your medication is the only thing that will help you…focus on your own life.” FG5_M
(+) Hope, ambition	“I wish to get well so that I can go back to school because I have already started placing applications, I want to further my education and see myself achieving my goals, I so wish I can have that opportunity where I can see myself wearing my graduation colours.” FG10_F

Recordings and notes of focus group sessions were transcribed verbatim by data collectors, deidentified, cross-checked for accuracy within a week of each session, and translated to English from isiZulu within 4 weeks. (−) denotes a disruptive concept and (+) denotes an ameliorating concept. ARV=antiretroviral. DRTB=drug-resistant tuberculosis. F=female. FG=focus group. M=male. MDR=multidrug-resistant. TB=tuberculosis.

## Research in context

### Evidence before this study

Current evidence on patient-related challenges with new, all-oral treatment regimens for drug-resistant tuberculosis (DRTB) focuses on access to new drugs such as bedaquiline and delamanid, and their side-effects or toxicities, or both. There is no documentation of wider and potentially persisting social and therapeutic complexities of treatment, how these complexities might change over the treatment course or among people with HIV co-infection, or both. A small study from South Africa (eight patients) delineated temporal challenges with DRTB treatment; however, most participants were receiving older injectable-based regimens and their HIV status was not reported. Our assessment of the evidence before this study is based on PubMed, EMBASE, and Google Scholar searches from database inception to July 31, 2020, using combinations of search terms “bedaquiline”, “delamanid”, “pretomanid”, “linezolid”, “patient”, “perception”, “experience”, “perspective”, “challenge”, “tuberculosis”, “drug-resistant tuberculosis”, and “HIV”. No language restrictions were applied to these searches.

### Added value of this study

This study fills a gap in the evidence around patient acceptability for new DRTB treatment regimens, particularly in the context of HIV co-infection. Drawing on in-depth qualitative methods and a conceptual framework for chronic illness, this study chronologises challenges and coping strategies among patients receiving bedaquiline-based treatment for multi DRTB or extensively DRTB and concurrent antiretroviral treatment in the high-burden setting of KwaZulu-Natal, South Africa. The study delineates patients’ needs during four critical stages in the DRTB care cascade—diagnosis, treatment initiation, discharge into the community, and treatment continuity—and provides novel insights into the means by which DRTB care and treatment can give rise to challenges for patients compared with HIV care and treatment during the period of comorbidity.

### Implications of all the available evidence

The needs and challenges of patients co-infected with DRTB and HIV fluctuate over the course of DRTB treatment, with the greatest gaps in information and support being at the stage of DRTB diagnosis, discharge from hospital, and treatment continuity in the community. The roll-out of new DRTB drugs and efforts to decentralise treatment into community settings should be supported with commensurate investments into holistic and reliable institutional, community, and home-based supports at key stages to enable patient-centredness (eg, DRTB treatment literacy and counselling for patients, families, and primary care providers; peer-based and faith-based patient interventions; social protection for DRTB-affected households; decentralised, destigmatising, and accessible DRTB care). DRTB programmes might benefit from adopting innovative approaches to service delivery used within other infectious disease programmes (eg, HIV and emerging programmes for COVID-19), even while adhering to crucial public health standards for infection control.
