# Supplementary material for: Dynamic needs and challenges of people with drug-resistant tuberculosis and HIV in South Africa: a qualitative study
Source: Lancet Glob Health. Author manuscript; Available in PMC 2021 Apr 1. (PMC8009302; doi:10.1016/S2214-109X(20)30548-9)
Supplement: 1 [file NIHMS1684398-supplement-1.pdf]

THE LANCET
Global Health

Supplementary appendix

This translation in isiZulu was submitted by the authors and we reproduce it as supplied. It has not been peer reviewed. *The Lancet*'s editorial processes have only been applied to the original in English, which should serve as reference for this manuscript.

Lokhu kuhumusha ngesiZulu kwethulwe ngababhali futhi siyikhiqiza kabusha njengoba kuhlinzekiwe. Akuzange kubuyekezwe ontanga. Izinqubo zokuhlela zeLancet zisetshenziswe kuphela koqobo lwesiNgisi, okufanele lusebenze njengesethenjwa salo mbhalo.

Supplement to: Daftary A, Mondal S, Zelnick J, et al. Dynamic needs and challenges of people with drug-resistant tuberculosis and HIV in South Africa: a qualitative study. *Lancet Glob Health* 2021; **9:** e479–88.

Dynamic needs and challenges of people with drug-resistant tuberculosis and HIV in South Africa: a qualitative study

Izidingo nezinselelo ezinamandla zabantu abanokumelana nemithi yesifo sofuba ne-HIV eNingizimu Afrika: ucwaningo lwekhwalithethivu

Amrita Daftary, Shinjini Mondal, Jennifer Zelnick, Gerald Friedland, Boitumelo Seepamore, Resha Boodhram, K Rivet Amico, Nesri Padayatchi, Max R O'Donnell

Summary/ Iqoqa

Introduction/ Isendulelo: Buncane ubufakazi bezinga lokwamukela kweziguli ezinikwa unakekelo lwesifo sofuba (TB) esiphika imishanguzo (DRTB) lapho kubhekenwe nohlelo lokwelashwa olusha kanye nokutheleleka ngasikhathi sinye nge-HIV. Sihlose ukuchaza okuzwiwa yiziguli ngonakekelo lwe-DRTB-HIV ezigulini ezisesifundazweni saKwaZuluNatali, eNingizimu Afrika.

Methods/ Itulokwenza: Kulolu cwaningo oluyikhwalithethivu olusebenzisa uhlaka luka-Bury lwezigulo ezingelapheki, sihlele izigungu ezingu-13 esibhedlela esikhulu sisebenzisa iziguli ezingama-55 ezihaqwe kanyekanye yi-DTRB kanye ne-HIV (abesifazane 28, abesilisa 27) ebezilashelwa i-DTRB ngemishanguzo emisha eyenziwe nge-bedaquiline, isetshenziswa umkanye nohlelo lokwelapha ngokushanguza kokuthithibalisa igciwane. Iziguli ezifanelekile ukuba socwaningweni bekungezikhulile ezinganika igunya (ubudala> 18 iminyaka) futhi kuqinisekisiwe ukuhaqeka kwazo nge-DTRB ne-HIV ebezibhaliswe kwiPRAXIS yocwaningo zinamasonto amabili ziqalise ukwelashelwa i-DTRB ngemishanguzo eyenziwe nge-bedaquiline. Abazibandakanyile babethathwe equlwini lePRAXIS ukuba bazibandakanye nesigungu ngokususela esikhathini asebenaso besekulashelweni i-DRTB:kusesemanzi (2-6 amasontongemva kokuqala ukwelashwa), sekumaphakathi (2-6 izinyanga ngemva kokukhululwa esibhedlela kumbe ngemva kokuqaliswa ukwelashwa uma engazange angeniswe esibhedlela), nangesikade (>6 izinyanga ngemva kokuqala ukwelashwa). Izigungu zocwaningo bezixoxa ngolimi lesiZulu, zaqoshwa, futhi zahunyushelwa esiNgisini emasontweni amane. Abazibandakanyile babebuzwa ngabahlangabezane nakho ekunakekelweni nasekulashelweni iDRTB ne-HIV, ulwazi oluyikhwalithethivu lahlelwa ngamakhodi nangezindikimba lwacutshungulwa futhi kanjalo.0.

Findings/ Okutholakele: Kusukela ngoNdasa ka2017, kuya kuNhlangualana 2018, kwahlonzwa izingqinamba ezikhethekile ezigabeni ezine ezisemqoka zonakekelo lwe-DRTB: Ukuhlola isigulo, okubonakala ngokungeniswa esibhedlela, ukunqindwa ekuphileni indlela yokuphila evamile, ukufakwa uphawu lobubi okuhleliwe futhi, ezigulini eseziphile isikhathi eside ne-HIV, ukuphazamiseka okusha; ukuqalisa ukwelashwa, okuvezwa yizidubulo, ukwahlukaniswa nabanye, kanye nokuqheliswa emphakathini; ukukhishwa esibhedlela, okuvezwa ngukuphazamiseka kwempilo yasekuhlaleni nesimo sokwelapheka esingusehla senyuka; kanjalo nenqubeko, okuvezwa yizinselelo eziya ngokujula zezomnotho ekuphileni yize kukhona ukwelulama kwezempilo. Izithuba zokuhlonzwa ngokuhlolwa nokukhululwa esibhedlela uye emphakathini yizo ngqo ebezinzima . Ulwazi ngemishanguzo kanye nokuphuthuma ekuthathweni kwezinqumo bekuligebe elilokhu livele njalo. Iminyombo yethunzi elimnyama ibilokhu ithi qhamthushu ndawo zonke phakathi kwesibhedlela nomphakathi. Ukuphikelela bekwakheka ezigulini ngokuzixhumanisa nontanga, ukuzihlukanisa nabanye, ukusimamiseka ngokwezezimali nezidingo zokuphila, kanjalo nokugxilisa umqondo ekwelulameni.

Interpretation/ Isihumusho: Abantu abane-DRTB kanye ne-HIV babhekana nezimo eziphazamisayo neziguqula impilo. Ukuntuleka kolwazi, ukuphuthuma, kanye nokuvikeleka komphakathi onakekelweni nasekwelashweni kwe-DRTB kudala izinselela ezibanzi ezigulini kunalezo ababhekana nazo nge-HIV kuphela. Amatulo okunakekela i-DRTB angagxilisiwe endaweni yinye, angawomphakathi, awokubambisana kontanga, nahlukanisiwe abenza kube ngcono futhi esiza ukukhuphula okwethenjiswa yizinhlelo zokwelapha ezintsha.

Funding/Uxhaso: US National Institutes of Health.

Copyright © 2021 The Author(s). Published by Elsevier Ltd. This is an Open Access article under the CC BY-NC-ND

Research in context/ Ucwaningo esimeni

Evidence before this study/ Ubufakazi ngaphambi kwalolu cwaningo

Ubufakazi bamanje ngezinselelo ezihlobene neziguli ezinesifo esisha, imishanguzo yokwelashwa yonke yesifo sofuba esingazweli emthini (I-DRTB) igxile ekufinyeleleni emithini emisha efana ne-bedaquiline kanye ne-delamanid, nemiphumela yayo emibi noma ubuthi, noma kokubili. Ayikho imibhalo yezinkinga zokwelashwa ezibanzi futhi ezingaqhubeka nokuphikelela nezinkinga zokwelashwa, ukuthi zingashintsha kanjani kule nkambo yokwelashwa noma phakathi kwabantu abane-HIV, noma lezifo zombili. Isifundo esincane ezivela eNingizimu Afrika (iziguli eziwu-8) zichaze okwesikhashana izinselelo zokwelashwa kwe-DRTB; noma kunjalo, iningi labahlanganyeli babethola izinhlobo zemithi emidala yomjovo kanye neimo sabo seHIV asizange sibikwe. Ukuhlolwa kwethu kobufakazi phambilini kwalolu cwaningo lususelwa ku-PubMed, EMBASE, naku-Google Scholar. Ukusesha kusuka ekuqaleni kwedatha kuya kuNtulikazi 31, 2020. Kusetshenziswe inhlanganisela yamagama osesho: “bedaquiline”, “delamanid”, "Pretomanid", "linezolid", "isiguli", "ukuqonda", "isipiliyoni", “Umbono”, “inselelo”, “isifo sofuba”, “ukumelana nemithi kwisifo sofuba ”, kanye ne-“ HIV ”. Ayikho imikhawulo yolimi esetshenzisiwe kulokhu kuseshwa.

Added value of this study/ Inani locwaningo

Lolu cwaningo luvala isikhala esikhona ebufakazini obuzungeze isiguli ngokwamukeleka kwemithi yokwelashwa emisha ye-DRTB, ikakhulukazi kumongo wokuthelela futhi nge-HIV. Ukuvezangokujulile izindlela zekhwalithathive kanye nohlaka lokugula okungapheli, lolu cwaningo lubala izikhathi nezinselelo nokubhekana namasu phakathi kweziguli ezithola i-bedaquiline ekwelashweni kwe-DRTB kanye nemishanguzo yokuthithibalisa igciwane leHIV endaweni enomthwalo omkhulu we DRTB ya KwaZulu-Natali, eNingizimu Afrika. Ucwaningo luchaza izidingo zeziguli phakathi nezigaba ezine ezibucayi ekwehlelweni kokunakekelwa kwe-DRTB—ukuxilongwa, ukuqala ukwelashwa, ukuphumela emphakathini, nokuqhubeka kokwelashwa —futhi kunikeza imininingwane emisha mayelana nokusho ukuthi ukunakekelwa nokwelashwa kwe-DRTB kungaveza izinselelo zeziguli uma kuqhathaniswa nokunakekelwa nokwelashwa kwe-HIV.

Implications of all the available evidence/ Imiphumela yabo bonke ubufakazi obukhona

Izidingo nezinselelo zeziguli ezitheleleke nge-DRTB neHIV ziyashintshashintsha phakathi kokwelashwa kwe-DRTB, ezikhaleni ezikhulu kakhulu kulwazi nokusekelwa ezisesigabeni sokuxilongwa kwe-DRTB, ukuphuma esibhedlela, kanye nokwelashelwa emphakathini. Ukukhishwa kwemishanguzo emisha ye-DRTB kanye nemizamo yokwelashelwa ezindaweni zomphakathi kufanele zixhaswe ngokutshalwa kwemali ezikhungweni eziphelele futhi ezinokwethenjelwa, umphakathi, nokusekelwa kwasekhaya okunezinto ezisemqoka ezigxila esigulini (isb., ukufunda ngokwelashwa kwe-DRTB nokwelulekwa kweziguli, imindeni, kanye nabahlinzeki bokunakekelwa okuyisisekelo; ukusekelwa kontanga nokukholwa, ukungenelela kweziguli; ukuvikela imindeni ethinteke nge DRTB; ukuvikela ukwenziwa inhlekisa, kanye nokunakekelwa kwe-DRTB okufinyelelekayo). Izinhlelo ze-DRTB zingahlomula ngokusebenzisa izindlela ezintsha ezisetshenziswa ngezinye izinhlelo zezifo ezithathelwanayo (isib. i-HIV kanye nokuqhamukayo njenge COVID-19), kulokhu kusetshenzwa ngezinga eliphakeme kakhulu eligwema ukutheleleka komphakathi.

Introduction/ Isingeniso

Ubunzima bokuhlonza isigulo nokusishanguza, ukuphatheka, kanye nokufa okweyamaniswa nofuba oluphika imishanguzo (i-DRTB) kulenza ufuba lolu lube yisimo esilukhuni salesi sifo. Unyaka nonyaka cishe yisigama sesigidi abantu abahaqwa yilolu fuba oluphika imishanguzo eminingi (MDRTB) olungezweli emishanguzweni yokuqala yokulwa nofuba i-isoniazid kanye ne-rifampicin. U-10% weziguli ngaphezu kwalokhu kawuzweli kwi-fluoroquinolone nesigaba sesibili semijovo futhi zinofuba oluphika imishanguzo ngokudephile (XDRTB)(1). Imiphumela yempumelelo ekulashweni kwe-DRTB iphansi kakhulu(28-52%)(1) Isithuba sokwelashwa –okuze kube yimanje besisuka ezinyangeni ezingu-18-24 – izidubulo ezivezwa yimithi, ukuhlaliswa ngawedwana, kanye nobunzima kwezezimali, emqondweni, kanye nokwezenhlalo okuhambisana nalokho kuyizithiyo ezisemqoka ezigulini(2).

ENingizimu Afrika kubikwa izigameko ezintsha zokutheleleka ngofuba kubantu abangaphezulu kuka-322 000, futhi u-4% walezi zigameko ngezofuba oluphika imishanguzo. Cishe u-60% weziguli ezinofuba ziphila futhi ne-HIV.1 Ngo 2015, i-National Tubercolisi Program iqale ukufaka uhlelo lokwelapha olufushane lwezinyanga ezingu-9-12 kwabane-MBRTB (izinyanga ezingu-18 kwabane-XDRTB], okuthe kancane kancane kwaholela ezinhlelweni zokwelapha lapho isigaba sesibili semijovo sesithathelwe indawo yimishanguzo ephuzwayo, ikakhulukazi i-bedaquiline(3,4). Ulwazi olusezincwadini olumayelana nokwemukela kweziguli izinhlelo zokwelapha i-DRTB ezintsha luseyivelakancane. Ukwelapha okuxube imishanguzo eminingi kusathatha isikhathi eside, kanjalo nezidingo zokwelapha zeziguli eziphila ne-HIV kazibikiwe kahle. Ucwaningo oluchaza izinselele zokwelashwa kwe-DRTB lusinika amazwibela akha phezulu kodwa kaluvamile ukuhlola izinguquko ezizokuba nomthelela onakekelweni olugxile ezigulini (2,5-7).

Ukuze sibhekane nalamagebe futhi sibeke endleni amangenelelo, sichaza lapha okuzwiwa yiziguli uma ngonakekelo nokwelashwa kwe-DRTB okutholwa ziguli ezsehlelweni olusha lokwelapha nokwelashwa ngemishanguzo yokuthithibalisa igciwane.

Methods/ Izindlela

Study design/ Indlela yocwaningo

Lolu cwaningo lwekhwalithethivu luthathisele kwinjulalwazi kaBury yokuphazamiseka kweminininingwane yobunjalo babantu, neyathuthukiselwa ukuhlola izinguquko ezibucayo nokuphazamiseka okuba kokulindelekile, kwizihlonzi, ebudlelwaneni, ezinhlelweni kanye nasezinhlakeni zempilo yemihla ngemihla kubantu abaphila nezifo ezixhwalisauyo ezingalapheki. Nezinhlelo ezisetshenziswa yiziguli uma zilungisa iziphazamiso ukuze zibuyelwe indlela ezizihlonza ngayo nezinga lempilo (8). Ukudepha kokwelashwa kwe-DRTB, kuhambisana ne-HIV, kukwenza kube yisiphazamiso empilweni yeziguli yangemihla ngamihla. Nokuyingakho uhlaka luka-Bury belufanelekile ukuze lubeke ngokulandelisa izikhathi izimo ezibhekane neziguli

Ukwelashwa kwe-DRTB-HIV esibhedlela esikhulu sasesifundazweni saKwaZulu-Natal. Lolu cwaningo lwe-PRAXIS lugunyazwe yikomithi elibheka isimilo socwaningo eNyuvesi YaKwaZulu-Natali (BE242/16) ne-Columbia University (IRB-AAAQ5753).

Participants/ Ababambiqhaza

Kusukela ngoLwezi, 2016 kuya kuNhlolanja, 2018, abadala abasezingeni lokuzivumela (>18 years) abangama-200 abaqinisekisiwe ukuthi bane-DRTB ne-HIV bazibhalisa ocwaningweni benamasonto amabili beqalise ukwelashelwa i-DRTB ngemishanguzo ene-bedaquiline njengokomhlahlandlela kazwelonke wezobudlewano, izinhlelo nezinhlaka zempilo yangemihla.

Kwi-PRAXIS, iziguli zingeniswe ezibhedlelwa zase zikhululwa ngemva kokuguqulwa isikompilo ngokubona kodokotela abasebenza kuleyo ndawo futhi bathole nemishanguzo yokuthithibalisa igciwane njengokwesidingo sezinga lonakekelo kuhambisana namatulokwenza amileyo ophenyo oluyikhwalithethivu (9-11). Lolu cwaningo beluzinziswe ezigungwini ezihlelelwe ukuhlonza zocwaningo lwePRAXIS (Promoting Engagement in the DRTB- 55 administered. HIV Care Continuum in South Africa, NCT03162107) ucwaningo oluxube amatulokwenza lokuhlola ukulandelwa ukwelashelwa ngaphandle ngokugcwele kwakungavamile ngalesi sikhathi sokuqalisa ukukhishwa kwe-bedaquiline. Ngesikhahi sokulashwa kweziguli zingaphakathi esibheldlela, iziguli bezizithathela imithi yazo kepha ngaphansi kwehlo noma kokuholwa ngabasebenzi basesibhedlela. Lapho sebekhishwa, babe sebezolanda imithi kuleso sizinda zinyanga zonke, sebezithathela ngokwabo. Usizo lwe-DRTB nolwe-HIV belutholakala emitholampilo eyahlukene nakuba kusesibhedlela esisodwa. Imishanguzo iyaguqulwa kusukwa esikalini somxube omile kuya kumxube oyisiikalo esingamisiwe, ukuze kugwenywe ukuxubana kweizinhlayiya zemithi ne-bedaquili (12).

Ngemva kwezinyanga ezi-5 ngemva kokuqala kocwaningo lwePRAXIS, umsebenzi wasocwaningweni umeme abazibandakanyayo abanye be-PRAXIS ukuze babe yingxenye yesigungu esakhiwe ngenxa yesikhathi asebenaso belashwa i-DRTB; kusesemanzi(2-6 izinyanga emva kokuqalisa ukwelashwa), sekumaphakathi(2-6 izinyanga emva kokukhululwa noma kokuqaliswa imishanguzo uma ungangeniswanga esibhedlela), sekuhlwile(>6 izinyanga ngemva kokuqaliswa ukwelashwa)

Izigaba zokubhalisa zishiywe zivulelekile ngoba abazibandakanyayo bebengaphansi kwezinhlelo zokwelashwa ezahlukene kanye nezithuba zokungeniswa ezibhedlela ezahlukene, futhi nathi sisophe ukuchaza izigameko noma izigaba zonakekelo abazibone zifanele. Ukubhalisa bekuholwa umgomo wokuhlukanisana (13) obekulendeleke ukuba utholakale ngezigungu ezingu-12.

Izigungu zifakwe ohlelweni lwefomu lwemvume ehlahlamilisekile ebhaliwe ukuze zibe seqlwini elizobheka. Kwatholakala imvume ngomlomo kumuntu ngamunye ozibandakanye kulezi zigungu, banikwa nethuba lokubuza imibuzo nokulandula ukuzibandakanya ngale kokuthikameza unakekelo lwezempilo abalutholayo noma isimo sabo ocwaningweni. ABazibandakanye ezigungwini bebethola okokusula umlomo kanye no-ZAR150 (US$12); abalashelwa ngaphandle kwesikhungo banikwe imali yokugibela.

Procedures/ Inqubo

Izigungu ezingu-14 zibanjwe ngesiZulu egunjini elingasese elishaya kahle umoya kuso isibhedlela esikhulu, futhi bekuqoshwa izinkulumo. Umdidiyeli ubebuza imibuzo ethathisela ohlakeni olulethwa zigungu esigabeni ngasinye (isithombe 1). Umhloli yena usize ngokuhlela izinhlelo nokuloba amazwibela. Bobabili umdidiyeli nomhloli bebengabasebenzi basocwaningweni abaqeqeshwe kabanzi ekubambeni izingxoxomibuzo zekhwalithethivu nokudidiyela izingxoxo zamaqembu futhi kababandakanyeki ekunakekelweni kweziguli nasekulandeliseni imithetho. Ukubekeka kwamagama emibuzweni nokuyilandelanisa kanye nokususa izingxoxo (ukuhlokoloza ukuze ukhulume) bekulukhu kuguquguqulwa ukuze kufanele isimo samaqembu abeyilokhu eguquguquka njalo. Abazabandikanyayo bebekhuthazwa ukuba babelana ngezimo ababhekane nazo nemibuno ehlukile kunaleyo ebekiwe. Kwakhiwe amaqenjana anamalungu azibandakanyayo angu-4-8 ukuze kugwemeke kahle ukuthelelana futhi banikwe amagama okungewona awabo kanye nokuhlelelwe ukuswabulukisa isimo, ukujwayelana kwamalungu kanye nokwethembana. Bekulindelekile ukuba abanye abazibandakanyayo kungenzeka bangatholakali ngezinye izikhawu zokuhlangana, ngemva kokuba sebevumile ukuba yingxenye.

Okuqoshiwe namazwibela abhaliwe kube sekubhalwa phansi izwi nezwi ngabaqoqilwazi, kwasuswa izihlonzi, kwaqinisekiswa ngokubhekisiselana ubuqiniso kungakapheli iviki esigungunwini ngasinye, futhi kube sekuhunyushelwa esiNgisini emasontweni amane kuqashelwe kahle izingathekiso kanye nezinye izinkomba ezijulile zolimi. Ukuhlaziywa kokuqala bekuphindaphinda, kuhambisana nokuqoqwa kolwazi, ukuze kucutshungulwe kahle ukuphenyakanye nokuhlola ukuhlukanisa (ukuhlukanisa kutholakale esigungwini se-11) ngendlela yokuyalela ngokuxoxisana nabaqoqilwazi nokubuyekeza amazwibela aqoqwe ezigungwini, imibhalo yokuqoshiwe, kanye nemisindo eqoshiwe. Ulwazi lonke lwafakwa kuNVivo 12 (QSR International) ukuze luhleleke ngokwezindikimba.[5,16,17] Kwenziwa futhi uhlelo lokugabazisa ngezinombolo, okushiya isikhala sokuba kwenziwe uhlaziyo lokuqalisa; ukufaka njenge-memo okwenza kube nokuqhathanisa kwezinga eliphezulu.

Amagabelo ezinombolo ahlukaniswe ngaphansi kwezihloko ezinkulu, kusetshenziswa amatulo avuna izingxoxo ukuze kuhlonzwe futhi kubuzwe kabanzi amaphethini, kuhlolwe isimo, futhi kuzuzwe ukuhlaluka kahle kwezihloko .[15,16] Izihloko ezaphuma lapho zamiswa eceleni kwezinhlaka zokunakekela ezitholakale zibalulekile kwabazibandakanyayo. Uhlaka lukaBury[8,11] luholelele ukuvezwa kakhudlulwana kwezindikimba futhi u-AD noBS kwenziwa uhlaziyo lokuqala lokuhluza. Ukugabazisa ngezinombolo kwenziwa ngokuzimela ngu-AD no-SM, baphinde bahlangana ukuze baphucule ukuthembeka kwalolo hlelo. U-JZ no-GF babeyingxenye ngokulandelana kwabo ekuthuthukiseni izihloko nezindikimba. U-BS waqhubeka nokucubungula izinhlaka zezolimi namagama ngokuxoxisana nabaqoqilwazi ngoba bona bebelukhuluma ngokugcwele ulimi lwesiNgisi nesiZulu futhi beyingxenye yohlelo locwaningo. Amasu aqhumukayo acutshungulwa ngokuwabuyekeza nangokohlelo lokuxoxisana nethimba lonke ukuze kuqikelelwe ukuthembeka kwawo.

Role of the funding source/ ___indima yomthombo wezimali________

Umxhasi wocwaningo ubengenaqhaza ohlelweni, ekuqoqweni kolwazi, ekuhlaziyeni ulwazi, ekuhumusheni ulwazi kumbe ekubhaleni umbiko ngocwaningo.

Results/ _Imiphumela__________

Kusukela ngoNdasa, 2017, kuya kuNhlangulana , 2018, kuphothulwe neziguli ezikhethekile ezingama-55 ezine-DRTB ne-HIV (30 abesifazane ezigungwini eziyisikhombisa, 25 abesilisa ezigungwini eziyisithupha) nezigungu (abane bonakekelo olusesemanzi, abathathu banokekelo oselumaphakathi, abayisithupha bonakekelo oseluqongile), ngasinye sinabazibandakanyayo aba-3-7 bobulili obubodwa). Isithuba sesigungu besivame ukuba yimizuzu eli-110 (maphakathi kwengu65-135). Iziguli zonke bezihlolwe zahlonzwa njengeziphila ne-HIV futhi zithatha imishanguzo yokuthithibalisa igciwane andukuba zihlonzw i-DRTB futhi ziqalise ukuthatha i-bedaquiline (table 1). Zonke iziguli ezithintiwe zivumile ukuzibandakanya, nakuba esisodwa noma ezimbili esigungqini ngasinye besinokungabi khona ngoba zinamanye amadinga noma zingazizwa kahle. Iziguli ezine zijoyine isigungu sesibili ngesikhathi sezidlulelwa ezingeni elehlukile lokwelashwa kanti izimpendulo zazo bezingehlukanga kwezalabo abazibandakanye nabo. I-HIV ne-DRTB ziyiguqulile impilo yemihla kube indlela yobubona kwabazibandakanyo, lokhu okuthikameze ukuzihlonza kwabo, imizimba (inyama nomqondo), ubudlelwano basemphakathini kanye nezezimali, kuhambisana nokuguquguquka okwesikhashana ngokuhamba kwesikhathi (isigaba sokwelashwa) kanye nesizinda (esibhedlela noma ekhaya). Abazibandakanyayo bamenywe ngokubuka ngokuhumusha isikhathi asebenaso ekulashelweni i-DRTB. Nokho, okuzekwe ngabazibandakanyayo yikho okusiholele ekuhleleni izimo ezibucayi noma izigameko abazibona njengeziphazamisayo noma ezidambisayo ezigabeni ezine ezigibelana nohlelo oluvamile lwesikhathi sokugula nge-DRTB, kusukela esikhathini sokuhlonzwa (figure 2). Okwehlukile okuhambisana nobulili babazibandakanyayol; noma ukutheleleka kwabo kuyaqashelwa lapho kuvela. Okukhethekile okucashunwe kubo kanye nokubaluleka kwako kubukwe ngokusobala (table 2).

Isigaba 1: Ukuhlonza isifo nokungeniswa esibhedlela—umshikashika wokuqala

Esigabeni sokuqala, ukuhlonzelwa i-DRTB kusho ukuhlolwa kaningi ezikhungweni eziningi, okuvame kuhambisana nokubambezeleka. Izindaba zokugula zadluliselwa ezigulini ngephimbo elisemqoka neliphuthumayo, futhi ziguli eziningi zibe nokuthuka okukabili uma betshelwa ukuthi bazokungeniswa esibhedlela esikhethekile isikhathi esingebalulwe. Iziguli bezigula kakhulu ngaleso sikhathi, futhi ukuhlonzwa kuholele ekunikweni unakekelo lwezempilo olusheshayo. Ukuba solubala nokwethuka iziguli ebezithathwa ngakho kuzishiye zizwa zijongiwe futhi zenyanywa. Iziguli kufaneleke ukuba zifake isifonyo, zime ojengeni olukhethekile, zime ngaphandle kwemitholampilo noma ziyiswe emawodini ahlukene. Kube nencazelo encane enikwe izigulu futhi zingenalo nekhethelo ngodaba lokulaliswa ezibhedlela, okuzishiye zididekile futhi ziphazamisekile. Kwakungamangazi ukuthi ezinye zazo zala ukuqala (ngamagama azo zaphulukundlela) ukudla imishanguzo ukuze beyobhekana nezinto eziyimpendulelo yempilo yabo. Ngenxa yokuba ukuhlonzelwa i-DRTB kuluhlelo olude, iziguli zikuvezile kumuntu oyedwa, okuvame ukuba ngumuntu oyisethenjwa wasemndenini, ukuze basizwe ngonakekelo lwasemndenini noma ukuhanjiswa. Iziguli ziveze imininingwane embalwa ngokungemukeli imishanguzo kofuba. Ulwazi oluncane iziguli ebezinalo nge-DRTB – okungukuthi i-DRTB iyathelelana kakhulu nokuthi ingakubulala – lwenze ukuba kube nokwesaba. Iziguli ezimbalwa zikuqaphelile ukuziqhelisa kwabantu ngokushesha emva kokuba seziveze ukuba banalokhu kugula, ngamagama abo “i-TB enkulu”, nokuyindlela evamile okubizwa ngayo i-DRTB, nokuba qede amandla ukuba baqhubeke nokuzidalula, iziguli zisebenzise izizathu ezingamampunge nje ukuze zishiye emesebenzini noma emiphakathini yazo. Izimoto zikahulumeni nonesi

abafake izifonyo , nokhom bebezihambela amakhaya ezinye zeziguli bebashiya bedengwane phambi kweso lomphakathi. Ufuba luvame ukuba luphawu lwe-HIV, lokhu obekubhangqa ukucwaswa nokuthunazwa kwesithunzi semindeni yabo. Iziguli ezitholakale ne-HIV ngesikhathi zihlolelwa ufuba zikuthole kulula ukudalula isimo sazo sofuba, nakuba benolwazi olufushane yize benawo umbikosimo we-HIV; i-HIV yaziwa njengesigulo esingunaphakade nesikhandlanayo. Ngokwehlukile, iziguli ebeziphila ne-HIV isikhathi eside zikuthole kunzima ukudingida ukuhlonzwa okusha. Lezi ziguli bese zike zadulalula i-HIV kulabo ezixhumene nabo, ngesinye isikhathi eminyakeni engaphambili, zanqoba izinguquko ebudlelwaneni (obusobala, nobezoncansi), bajwayela imishanguzo, baze babanamandla emzimbeni ukubhekana nezinto eziyingcuphe yezempilo neyendlela abahlonzwa ngayo. Izidingo ezintsha zokwelashelwa i-DRTB, bezikhombisa ukuba yingozi kulokhu kubambeka kwesimo. Ukudalula nokwemukela i-DRTB bekunozinselele, kungakhathalekile ukuthi wahlonzwa nini njengophila ne-HIV, ngoba ukuhlonzelwa i-DRTB bekuhambisana nokuhlongeka kolwazi uma uqhathanisa nalolo lwazi olwatholakala ngesikhathi se-HIV. Iziguli ezingenawo umlando Wofuba (ebebengakwazi ukwenza umqondo ekuvumbukeni isifo eseyamaniswa nokungalandelwa kwemiyalelo yokwelashwa), labo abazizwe bengenathemba ngokwelashwa esikhathini esizayo (ngoba bake baba nofuba noma ukwelashelwa ufuba kwaphunza phambilini), kanye nalabo abazizwe benyanywa kumbe bengenakwesekwa yilano ababethembayo (ikakhulukazi omama abathile), futhi bebenobunzima ukwemukela ukuhlonzwa njengabane-DRTB kanye nokuthi kufanele bangeniswe esibhedlela.

Isigaba 2: Ukuqala ukushanguzwa—esigabeni sesibili sebevalelekile futhi behlungiwe

Iziguli zibike umuzwa wokukhululeka nokuzinza ekudleni kwawo imishanguzo yokuthithibalisa igciwane ngesikhathi beqalisa ukwelashelwa i-DRTB. Bakhononde ngokumelwa ngukuthatha amaphilisi amaningi njalo kunesikalo esisha esingaqondile sohlelo lokwelapha elingumxube wameshanguzo yokuthithibalisa igciwane, kusukela ephilisini elilodwa ngosuku kuya kwamabili kabili ngosuku. Nokho, lokhu kuthe akufane kancane namaphilisi iziguli ebeziwathathela i-DRTB, Lokhu kwenze umthamo wamaphilisi eziwathatha ngelanga abe ngu-22-30. Ngaphezulu kwalo mthwalo wamaphilisi, iziguli zibone izidubulo ziyinselele enkulu ezinyangeni ezintathi zokuqalisa ukwelashewa i-DRTB futhi, nangale kokuba lamaphilisi bawadla kanye kanye nemishanguzo yokuthithibalisa igciwane, kepha zonke izidubulo zithathwe njengezize nemishanguzo ye-DRTB. Isiyezi, ukuxhuxhuma kwesisu, ukwelunywa, ikhanda, ukhwantalala, kanye nokushaya ngamandla kwenhliziyo bekuyizinto okuvame ukukhonondwa ngazo, yize ukuphalaza nobuhlungu bemisipha namajoyinti bekuzwakala njengokuphazamisayo impela. Iziguli eziningi zikhononde ngokungazinzi kwesimo somqondo futhi kungathi badla izidakamizwa ezithile ezingo semthethweni. Izinguquko endleleni yokubona nasesimbaleni wesikhumba bezingavamile kodwa zibonwa njengezingafuneki kakhulu. Cishe ikota yeziguli ethole uhlelo lwesibili lwezinto ezitholakala ngokujova ze –DRTB (table 1) andukuba ziqalise i-bedaquiline futhi zikuthakasele ukuguqukela ohlelweni lokwelapha ngemithi yonke ephuzwayo, nokho sikhona isiguli esigcizelele ukuthi ukujova kunamandla. Lezi ziguli nazo zibike izidubulo zeminye imishanguzo kanye nomthwalo omkhulu wamaphilisi, eziningi zazo zinezidubulo zesikhathi eside edaleke noma imishanguzo sezayiyeka kudala (isib. Ukulahlekelwa ukuzwa ngezindlebe). Ngokungakhathalekile ngalo mlandu, izidubulo zenze iziguli ezimbalwa zacabanga ukuyiyeka imishanguzo futhi iziguli ezinye ezimbalwa zivumile ukuba zizenzele ezazo izinhlelo zokwenza sengathi ziwagwinyile amaphilisi ngisho unesi ephambi kwazo. Ukususwa empilweni evamile kanye nasebudlelwaneni kube

ngomunye umnyombo wokukhungatheka. Ngale kwemiyalezwana yocingo nokushayelwa ezikutholile , cishe zonke iziguli zivele zanqindwa impilo yazo yokuxhumana nabanye. Iziguli ezimbawa kuthe ngokuhamba kwesikhathi zakutusa ukufakwa esibhedlela ngesikhathi zisaxinwe ngukugula, ukuze zikwazi ukungena kahle ohlelweni lwezokwelashwa olusha. Nokho, ukuhlala okweluliwe bezingakuqondisisi ukuthi kungani. Iizguli zichithe izinsuku eziningi zingomashayandawonye, zininga ngokufa noma zibuka abanye befa zingenayo nento yokuzikhipha kulo mqondo, noma ukuzikhethela okunye, kumbe izindaba zakamuva ngendlela zona eziqhuba ngayo. Imindeni eminingi ibihlala kude, futhi izihlobo zibukeke zingenalo ilukuluku lokuvakashela iziguli ezinokuzithelela ngokugula. Iziguli bezingenayo enye indlela kunokuzizwa zilahliwe, zizizwe zidengwane. Ukukhathazeka ngokwezezimali kukhulile kulabo ababengabondli emakhaya. Bayingcosana abebethola imihlomulo yabaqashi noma izibonelelo zikahulumeni, obekusetshenziselwa izidingo zasendlini, kepha iziguli eziningi bezingatholi lusizo. Iziguli zithole ukwelulekwa mayelana ne-bedaquiline, kepha olunye ulwazi ngemishanguzo balufunde ngendlela nangokuyisebenzisa. Iziguli ebezinolwazi oluncane ngokwelulama kwazo, izingcuphe zemishanguzo, izinguquko emithini yazo, nokuphuma esibhedlela, bezinobunzima impela futhi lokhu zisole ngakho ukuntuleka kwaloyo omelet ukubaxhuma. Iziguli bezesaba ukuthi abahlinzeki bosizo bazibukela phansi kumbe bayazisola ngesifo esiziphethe, kumbe bebezikhandlekele futhi bengenaluzwelo. Ukucela ulwazi oluthe xaxa noma ukweseka okuthe thuthu (isib. Umsebenzi wokuhlaliseka) kuholele ekutheni iziguli zibizwe ngesicefe noma izimpimpi ezifuna ukufaka izinkonondo. Lokhu kushayisene nosizi abaluthola ekwelashweni ngemishanguzo yokuthithibalisa igciwane kubasebenzi ababebhekene ne-HIV. Ukuphazamiseka ngookwemizwa nasenyameni kwaleli zinga bekudanjiswa ngokuxhumana okuhle komhlinzeki wosizo nabasesimweni esifanayo lapho ulwazi, izeluleko, uzwelo, kanye nokubingelelana bebekwenziwa, lokhu kwenze iziguli zahlala zithatha kahle imishanguzo yazo. Ukwabelana ngosizi ababhekene nalo nabanye abagulayo kuzale ukuba babe lumbimbi futhi kuxoshe nesithukuthezi. Ukuthandaza, ukukholelwa nguNkulunkulu, nesidingo sokuvikela amalungu omndeni kwi-DRTB bekuyizikhuthazi ezisemqoka kakhulu ezigulini ezichaze lesi sikhathi njengesimnyama kakhulu empilweni yazo. Ekugcineni iziguli zikuqondisisile ukuthi ukwelashwa nemishanguzo yilona kuphela ithuba ezinalo ukuze zisinde futhi zigcine zisemukele isimo sazo

Isigaba 3: ukudedelwa uye ekhaya— ukukhululwa nokuphakama futhi kweziphazamiso

Esigabeni sesithathu, sezinezinyanga ezi-3-6 zelashelwa i-DRTB, iziguli zizwe imizimba yazo sengathi isiyamukela. Iziguli bezisebuthaka futhi izidubulo bezisolubala, nokho besezingasaveli njalo futhi zingasekho bucayi njengasekuqaleni kusaqalwa imishanguzo. Iziguli eziningi zidedeliwe (table 1), futhi zathakasela ukubuye zihlanganiswe nemindeni yazo. Nokho, eziningi zibhekane nobunzima bokuzijwayeza nohlelo olusha lwakenye indawo yokuhlala. Emasontweni angemangaki, ukujabula kwezihlobo ngasekuqaleni kuqale kwathathelwa indawo ngukungenami, ukungabekezeli, kanye kanye nokungasikholwa isimo sokuxhwala kwesiguli esilokhu siqhubeka kanye nungakwazi ukuba yingxenye yemisebenzi yasekhaya kanti zidla nezinto eziyivelakancane. Ukuvuka futhi kokulimala kwendlela isiguli esizibuka ngayo kanye nokuphazamiseka kobudlelwano nazo nalabo abakholwa ukuthi isikhathi esinzima se-DRTB sesidlulile. Ukunyonkolozwa kweziguli kukhulile ngezindlela zokuthi ziyagwenywa, ziyadelelwa, futhi kazitholi lusizo emndeneni nakulabo ezinobuhlobo nabo. Ukudalula ngokuvulelekile isimo sezempilo kwabomndeni kanye nasezithangamini zokuxhumana bekungavamile, kodwa bekunzima ezigulini ukuba

zikufihle ukungabonakali kwazo isikhathi eside noma ukuguquka kwendlela ezibukeka ngayo. Omakhelwano bazile ukuthi iziguli zinofuba olubucayi, futhi kuyaqagelwa ukuthi onofuba usuke ene-HIV. Nakuba bekungacaci ukuthi okuyikhona okuthunaza iziguli ngukuba nesisodwa noma zombilei lezi zigulo, bekukhona nje ubulukhuni obungachazeki maqondana nengcuphe yofuba – isib. Obaba abambalwa abebeke badalula isimompilo sabo se-HIV bebenqindiwe ithuba lokuzobona izingane zabo. Iziguli ngakho-ke bezilokhu zizihlunga ukuze zibe ngcono futhi zigweme ukuthelele abanye kanti futhi nacho ukuze bagcine ukuzihlonipha kwabo basuse nalento yokunyonkolozwa. Ukuzizwa uvalelekile usemphakathini wakho bekuyindlela yokuzihlunga bekukugcina uwedwa impela ngaphezu kwalokhu iziguli ezikuzwe sezisesibhedlela, ezinye iziguli zishintshe indawo yokuhlala zaya kulabo abebekhombisa ukuba nozwelo noma abanosiza olubambekayo. Iziguli ezisencane ziphelelwe yimpilo yazo yobungani; bakuthole kukhathaza nje ukuxhumana nabanye futhi beqonda ukuthi ezinye izenzo(isib, ukuphuza utshwala kungabeka amathuba okusinda engcupheni. Ubudlelwano bezothando obuqubuke ngesikhathi besesibhedlela buphelile ezigulini ezimbalwa ngesikhathi sezibuyele ekhaya, yize obuningi ubudlelwano balolu hlobo buphele sekuhlonzwe i-HIV. Amadoda ambalwa alahlekelwe ngumdlandla wezocansi. Badikilwe yizintombi noma badeleleke, ikakhulukazi ngabesifazane abasebancane, lokhu kube yingcuphe ebudodeni babo futhi kuhambisanaa nokungazinzi kwesimo sezimali.Iziguli ezimbalwa zikhulume zinenkumbula ngbuhle bempilo yasesibhedlela lapho bebexhumene nezinye iziguli, bethola ukunakwa ngesikhathi kuhlanganisa nokudla kanye nokunikwa imishanguzo, kanye nokuthola njalo ukwelulekwa. Abasekhaya bebengenalo uzwelo olufana nolwezinye iziguli esibhedlela futhi nokudla sekuyivelakancane ukuba kulungiswe, futhi kazisakhunjuzwa iziguli ngokuthatha amaphilisi. Osonhlalakahle basemphakathini bebengayejwayele i-DRTB. Imitholampilo eseduze idlulisele iziguli emuva esibhedlela esikhulu ukuze zithole unakekelo olukhethekile ngokushesha uma isimo sazo se-DRTB sesihlonziwe, okwenza iziguli zibhekane nokuhamba ngama-ambulense nokuqhinqa ebusuku esibhedlela behlalele izmo ezincane (njengekhanda). Iziguli bezisekuqhubekeni nokushanguzwa kwe-DRTB obesekubandakanya imishanguzo embalwa; nokho, umthwalo wamaphilisi ubusephezulu kuzona futhi ububiza emzimbeni uma kubalwa ukukhathala okungumbelebele, isithukuthezi, ukulahlekelwa wumgqumo abaseziwubambile esibhedlela. Kungekho muntu obheke inqubekela phambili yazo, iziguli ezimbalwa ziphuthwe ngukuthatha imishanguzo yazo ngesikhathi noma kwayikhona nje ukuyithatha.

Ukwesekwa ngokwemizwa nangezinto eziphathekayo ekhaya kusize ukunciiphisa ubunzima ezigulini. Lapho izihlobo zizwalane futhi zizibandakanya enqubweni yesiguli (isib. Bekhumbuzisa futhi beletha izidingo zezempilo),iziguli zizizwe zikhuthazekile ukuba zingathatha amanyathelo abonakalayo aya Ekupholeni (isib. Zidla okunempilo, zigwema utshwala, zithatha ngesikhathi amaphilisi). Ezinye iziguli zifake izicelo ngokuzwakalayo ukuba uhulumeni makabe nohlelo lwemibhidlango yokwazisa nge-DRTB, njengalokhu enza ngeNgculazi, ukuze akhuthaze ngezidingo zazo ngokubonakalayo.

Isigaba 4: Ukuqhubeka nemishanguzo—kasikakafiki esiphethweni

Esigabeni sesine, esibe yizinyanga eziyi-8-12 kuqaliswe ukwelashwa, iziguli eziningi bezizwa zisempilweni. Ukuthanda ukudla kanye nesisindo somzimba sibuyele endaweni yaso kanjalo nokuthatha amaphilisi kungene khaxa empilweni yemihla; nokho, ezinye izidubulo ziqhubekile nokuba yisithikamezo. Ukuphaphatheka kwesikhumba okubonakalayo kunqinde iziguli ukuba ziphume ekhaya. Ubuhlungu bamanyongo, ubunzima bokugxilisa umqondo, ubunzima bokuzwa, ukukhathaza komqondo kuvimbele

iziguli ukuba zenze imisebenzi yasekhaya futhi zikwazi nokuzenzela. Izidubulo ezizibekezelele ngesikhathi zibhekene nokufa noma ngesikhathi behleli nezinye iziguli manje besezingasemukeleki sampela. Ukuba buthaka osekuthathe isikhathi eside kube yisikhumbuzo ezigulini ukuthi bagula ngokubonakalayo futhi bayathelelana, okube yisizathu kwabanye sokuzigcina beqhelelene nabanye. Ukunqindeka ezimalini nako kube ngomunye umnyombo omkhulu wesinxe. Imihlomulo nezibonelelo ebezinomkhawulo bese ziphele kudala. Iziguli ezimbalwa zizizwe sezikulungele ukusebenza kodwa amathuba abeyivelakancane kanti nomlando wokuba ne-DRTB ububhekwe njengozothiya abaqashi. Amadoda athile azitholile izikorobho (njengoshayela amatekisi) kumbe aphelela ekwebeni ukuze babe nendlela yokungenisa imali, ngalokho babuyelwe ngukuhlonishwa, kepha lokhu kuthikameze ukulandela izinhlelo zokwelashwa kanye namadinga ahlelwe nomtholampilo. Ukweyama ngabanye kwandile ezigulini, okwandise ukuzizwa zingelutho futhi zidengwane ekuthunazweni isthunzi, njengokulahlekelwa yizwi ezinqumweni zasekhaya kanye nezingxoxo ezithunazayo ngesimo sabo sezempilo. Ukwelashwa ngasekugcineni kwe-DRTB kuqhubekile nokuba yinselele ezigulini eziningi ebezingakabi nendlela yokusinga isikhathi esizayo lapho zisempilweni futhi zisebenza. Ukuphefumula kuze ngokusimama kwesimo sezimali (isib. Ukubonelelwa zihlobo noma ukusebenza) kanye nokululama ezidubulweni. Bekulokhu kuvezwe njalo ukulangazelela ukuthola imishanguzo ngendlela elula nengadali ukunyonkolozeka. Iziguli bezikulangazelela ukuthola unakekelo ezindaweni zomphakathi wazo, kepha zikuthokozela futhi ukuba ngasese kwezikhungo ezikude lapho bebekwazi ukubalekelana nokwahlulelwa ngomakhelwane kanye nabasebenzi bezempilo basendaweni. Iziguli ezizizwe sezingcono ziveze ukulangazelela uhlelo lapho bezoithola imishanguzo yabo ye-DRTB njengakwezinye izigulo, okuhlanganisa ne-HIV, emitholampilo noma emakhemesi ngale kokudinga unakekelo lokubonwa siqu. Ezinye iziguli zenze isinqumo esiphusile sokwamukela imishanguzo njengethuba lempilo, eliyisidingo sokubuyela empilweni yize kunokunyokolozeka nokuntuleka kokwesekwa. Abesifazane bebekhuthazwa ngabantwana babo kanye namanye amalukulunku abanawo. Amadoda aveze ukuthi isikhuthazi sawo sisekuyizibeni imibono yabanye bagxile kulokho okuyisidingo sempilo yabo.

Discussion/Ingxoxo

Lolu cwaningo lubeka isikhathi sobunzima beziguli nokubhekana nazo ngesikhathi sokwelashwa kwe-DRTB, kufakazela ukubaluleka kocwaningo lwesifo sofuba olugxile esigulini(2, 5-7, 17). Okutholakele kugxilwe ngokuhlukile kumongo wokulashwa, amaphilisi alapha izinhlobo ze-DRTB, bese ukhomba imikhawulo yobuchwepheshe, nentuthuko ekunciphiseni izinselelo zeziguli. Ithiyori yohlaka lokugula okungamahlalakhona lusiholele ekuqondeni ukuphazamiseka okungenzeka kwemicimbi ebucayi ngokunakekelwa kwe-DRTB. Futhi uncnywe ukungenelela okuphendula okususelwe ezimweni lapho abantu abane-DRTB bathola ukuthi bayashintsha nokukhuthazeka (umdwebo 2). Abesifazane, abesilisa, abazali, nabantu abanesifo sofuba esedlule noma esihlala isikhathi eside esinemiphumela emibi, babhekane nobunzima obuhlukile, okunye okuke kwabhalwa phambilini (2, 19, 20). Iziguli ezazike zaphila ngegciwane lesandulela ngculaza isikhathi eside ngaphambi kokutheleleka nge-DRTB futhi sezijwayele ukuphila nesifo esisodwa esingalapheki zabhekana nokuphazamiseka okusha. Ukuqiniswa komlando kubhaliwe kubantu abatholwe benezifo eziningi ezingalapheki (11). Lapha, sabona izinselelo ezidalwe yi-DRTB, ukwelashwa kweqa iziguli ezine-HIV, ngisho naphakathi kweziguli ezinokuphila isikhashana zine-

HIV, ngenxa yezimpawu ezinzima, ukwelashwa (isib. Wephilisi amaningi, imiphumela emibi), izidingo zokwelashwa (isib. ukulaliswa esibhedlela, ukuhlala wedwa), kanye nokuntula kolwazi nge-DRTB, imininingwane, ukwelulekwa, nokunakekelwa kwabahlinzeki. Lokhu okutholakele kugcizelela izinhlaka ezehlukene ze-HIV kanye nohlelo lokunakekelwa kwezempilo lwesifo sofuba, kanye nesidingo ukuhlanganisa izindlela zesiguli, imindeni nomphakathi ukuzibandakanya kuzilungiselela ezineDRTB ne HIV(7,21).

Ukuvela kwezinselelo nemiphumela yeziguli, ukusabela kanye nokubona kwabo kwakuphawuleka kubunjwe esikhundleni seziguli ze-DRTB, nalapho ukunakekelwa kutholwe khona. Lokhu kuqonda kungakhombisa ukungenelela okugxile ezigulini. Ukungabi nalwazi kwakusobala ekwelashweni kweDRTB kanye nemiphumela yalokhu kwazwakala ikakhulukazi ngesikhathi sokuxilongwa nokukhishwa esibhedlela. Ukungabi nokuzimela kwesiguli nokuzethemba kwakunzima ngesikhathi sokulaliswa esibhedlela, futhi lapho ukuzimela nokubuyela emplilweni ejwayelekile kwakulindelwe. Ukwelulekwa ngokwelashwa kweDRTB nokubandakanyeka kweziguli kungenzeka kuvale ezinye zalezi zikhala futhi kukhuthaze ukugcinwa ekunakekelweni (23). Izinhlelo zokusekela ontanga zinganciphisa inguquko uma umuntu esuka esibhedlela eya ekhaya, nokwandiswa ukwesekwa umphakathi nezinhlelo zokubuyela emsebenzini futhi kunganciphisa ukuthembela kakhulu kwabanye abantu(24).

Ukubonakaliswa kokucwaswa nakho kugudlukile. Ekhaya nasemphakathini, iziguli bezilokhu zingahlonishwa, zinganakwa futhi zingasizwa, kanti ohlelweni lwezempilo, zazivezwa ukutheleleka, zihluke kwezinye iziguli futhi ziyingozi; ukungathembi; futhi nokungakuxhumani kwabahlinzeki. Lokhu nakho kubhalwe ngaphambilini(17,25). Isifo sofuba sasivame ukuhlotshaniswa ne-HIV, okwandisa ukucwaswa(26,27). Sithole ukucwaswa kukhona, futhi ngaphandle kwalokho, ukwesekwa nezenhlalo kwakuyinto esizayo. Ukungenelela okusekelwe okholweni okuthuthukise izimpilo zalabo abaphila ne-HIV(29), kanye nosizo lwezezimali, kungaqinisa ukuzimela kweziguli. Ubhadane lwe-COVID-19 lungahlinzeka ngokuqhubekayo umfutho wokuxhasa okugxile emndenini, ukuqeda izinhlekelele, kanye nezindlela ezenzelwe amalungelo okunakekelwa kwezifo ezithathelwanayo (30).

Ukwelulela ukwelashwa kweDRTB ezindaweni ezikude nemizi yeziguli ekugcineni ingaxazulula ubunzima obuningi, kanye nemithi yonke ephuzwa ngomlomo (haayi imijovo) ingenza izinsizakalo zezempilo zomphakathi ezenziwe ngaphambilini zokunakekelwa emphakathini noma eduze nasekhaya(3, 31-33). Ukuze kuphumelele, ukwesekwa nendawo esekelayo iyadingeka emakhaya eziguli (indawo yokuhlala ezinikele, ukuvikeleka kokudla, izinsiza zokubambelela, nomndeni onolwazi, onozwelo) nasezigulini (amakhono amahle emitholampilo, okuxhumana, amathuba okusebenza, nokwazisa ngokululama kwe-DRTB hhayi izingozi kuphela). Ukunakekelwa okuhlukile, okuvame ukukhuthazwa ngaphakathi kwezinhlelo ze-HIV futhi ezisanda kuthunyelwa isifo sofuba(34), ingahlangabezana nezidingo zeziguli ezizinzile emitholampilo, futhi kingandisa ukunakwa kulabo abanezidingo nezinselelo ezinkulu.

Ukutadisha kwethu kunemikhawulo eminingana. Asikwazanga ukulandela abahlanganyeli ngokuhamba kwesikhathi, futhi sakhetha izigungu phezu kwezingxoxo ezizimele. Ngenxa yalokho, asizange sithwebule idatha yezinga lomuntu ngamunye. Noma kunjalo, amaqembu okugxila aya ngokuya abonwa njengokumbula ukudalulwa komuntu siqu ngezihloko ezizwelayo ezibucayi futhi kukhonjiswa ekwamukelweni kwabahlanganyeli maqondana nezehlakalo ezihlukumezayo uma kuqhathaniswa nokuziphatha okungamukeleki emphakathini (isb ukungabambeleli kahle emithini)(14,17,35). Izinselelo ezihlobene nokuhlala isikhathi eside ezibhedlela noma ukuphuza imishanguzo ye-HIV ama-antiretroviral ingahle ingasebenzi kangako ekunakekelweni kwabantu abahlala ezindaweni ezinezigameko eziphansi ze-

HIV, yize ukuqonda kokwesekwa okusekelwe emphakathini kungakha ukugxila ezigulini ze-TB ngokujwayelekile. Asizange sithwebule okuhlangenwe nakho okuhlobene nokufinyelela kwe-bedaquiline, noma ukuqeda ukwelashwa kwe-TB , noma ukubuyela ekunakekelweni kwe-HIV, okungaveza izinselelo ezintsha noma eziphikelelayo (5). Ukuqoqwa kwedatha ebanzi isikhathi eside kanye nophenyo olwenziwe eqenjini nalo alizange lifake imininingwane ngokuphuthelwa izikhathi zemithi njengoba kuxoxwe ngayo kumaqembu athile okugxila. kunalokho, lolu cwaningo luhlinzeka ngesisekelo esicebile lapho ukwedlulelwa kweziguli kudluliswa ukwelashwa kwe-DRTB nokwelashwa kwe-HIV kungalungiswa kangcono.

Ucwaningo lunikeze ukuqeqeshwa kwabahlinzeki besiza kanye nokunamathela emishanguzweni ehlukene kanye nokungenelela kokunciphisa ukucwaswa. Lolu cwaningo luqinisekisa isidingo sokupheleliswa nokwethenjelwa izisekelo zezikhungo zomphakathi nezasekhaya, kufaka phakathi ukufunda ezifundweni ezitholwe kwi-HIV, ukuhlangabezana nezidingo zesiguli zesikhathi eside nokusiza ekwandiseni isithembiso semithi emisha ye-DRTB.

Contributors/ Abanikeli

U-AD, uNP, no-MRO bahlela lolu cwaningo. U-AD wathuthukisa amathuluzi okufunda, ahola ukuhlaziywa, futhi wabhala okusalungiswa kokuqala kwefayela lombhalo wesandla. Ukuqoqwa kwemininingwane ngu-AD, BS, no-RB. U-SM no-AD babhala imininingwane, futhi bonke ababhali baba nesandla ekuhlaziyweni kwe-thematic. Bonke ababhali banokufinyelela okugcwele kuyo yonke idatha yokufunda futhi banesibopho sokuthembeka kwedatha nokuthembeka kokuhlaziywa. Bonke ababhali babhekele isinqumo sokuletha lo mbhalo wesandla ukuze ushicilelwe

Declaration of interests/ Isimemezelo sezintshisekelo

Asimemezeli ukuthi sinentshisekelo.

Data sharing/ Ukwabelana ngemininingwane

Imihlahlandlela yezingxoxo izotholakala kusuka Jan 1, 2021, kuya kuJan 1, 2024, lapho kucelwa umbhali u-adaftary@yorku.ca, ngokuya ngokuvunywa kwesicelo esihlongozwayo kwithimba lokufunda eligcwele. Imininingwane efanelekile yokutadisha yethulwe embhalweni wesandla namatafula. Ngenxa yemvelo echazayo, eminye imininingwane (kufaka phakathi idatha eqinisekisiwe) ngeke itholakale ukuvikela ababambiqhaza.

Acknowledgements/ Ukubonga

Lolu cwaningo luxhaswe yi-US National Institutes of Health (R01AI124413, abaphenyi abayinhloko u-MRO no-NP). Ukushicilelwa kwakungu kusekelwa Izilingo zomtholampilo zamazwe AseYurophu Namazwe Asathuthuka Isibonelelo sokubambisana (TMA2018SF-2467). Sibonga ababambiqhaza beziguli, abasebenzi besiza, noMnyango Wezempilo KwaZulu-Natali ngokuxhasa lolucwaningo; nabasebenzi baseCentre for the AIDS Programme of Research in South Africa, neColumbia University Irving Medical Center, NY, USA, ikakhulukazi uNokwanda Depargo, uNonkululeku Ngcobo, uKhethokuhle Nkosi, no-Allison Wolf ngokusiza ngemisebenzi yocwaningo.

References/ ___Izinkomba______

1. WHO. Global TB Report. Geneva: World Health Organization, 2019.

2. Thomas BE, Shanmugam P, Malaisamy M, et al. Psycho-Socio-Economic Issues Challenging Multidrug Resistant Tuberculosis Patients: A Systematic Review. *PloS one* 2016; **11**(1): e0147397.

3. Ndjeka N, Schnippel K, Master I, et al. High treatment success rate for multidrug-resistant and extensively drug-resistant tuberculosis using a bedaquiline-containing treatment regimen. *Eur Respir J* 2018; **52**(6).

4. WHO. Consolidated guideleines on drug-resistant tuberculosis treatment. Geneva: World Health Organization, 2019.

5. Furin J, Loveday M, Hlangu S, et al. "A very humiliating illness": a qualitative study of patient-centered Care for Rifampicin-Resistant Tuberculosis in South Africa. *BMC Public Health* 2020; **20**(1): 76.

6. Horter S, Stringer B, Greig J, et al. Where there is hope: a qualitative study examining patients' adherence to multi-drug resistant tuberculosis treatment in Karakalpakstan, Uzbekistan. *BMC Infect Dis* 2016; **16**: 362.

7. O'Donnell MR, Daftary A, Frick M, et al. Re-inventing adherence: toward a patient-centered model of care for drug-resistant tuberculosis and HIV. *Int J Tuberc Lung Dis* 2016; **20**(4): 430-4.

8. Bury M. Chronic illness as biographical disruption. *Sociology of Health & Illness* 1982; **4**(2): 167-82.

9. Brigaste MBT, Teh LA. The Battle Continues: An Interpretative Phenomenological Analysis of the Experiences of Multidrug-Resistant Tuberculosis (MDR-TB) Patients. *Psychological Studies* 2018; **63**(1): 9-18.

10. Calman L, Brunton L, Molassiotis A. Developing longitudinal qualitative designs: lessons learned and recommendations for health services research. *BMC Medical Research Methodology* 2013; **13**(1): 14.

11. Locock L, Ziébland S. Mike Bury: Biographical Disruption and Long-Term and Other Health Conditions. In: Collyer F, ed. The Palgrave Handbook of Social Theory in Health, Illness and Medicine. London: Palgrave Macmillan UK; 2015: 582-98.

12. O'Donnell MR, Padayatchi N, Daftary A, et al. Antiretroviral switching and bedaquiline treatment of drug-resistant tuberculosis HIV co-infection. *The lancet HIV* 2019; **6**(3): e201-e4.

13. Halcomb EJ, Gholizadeh L, DiGiacomo M, Phillips J, Davidson PM. Literature review: considerations in undertaking focus group research with culturally and linguistically diverse groups. *Journal of clinical nursing* 2007; **16**(6): 1000-11.

14. Morse JM, Barrett M, Mayan M, Olson K, Spiers J. Verification strategies for establishing reliability and validity in qualitative research. *Int J Qual Methods* 2008; **1**.

15. Nowell LS, Norris JM, White DE, Moules NJ. Thematic Analysis:Striving to Meet the Trustworthiness Criteria. *International Journal of Qualitative Methods* 2017; **16**(1): 1609406917733847.

16. Braun V, Clarke V. Using thematic analysis in psychology. *Qualitative Research in Psychology* 2006; **3**(2): 77-101.

17. Daftary A, Padayatchi N, O'Donnell M. Preferential adherence to antiretroviral therapy over tuberculosis treatment: a qualitative study of drug-resistant TB/HIV co-infected patients in South Africa. *Global public health* 2014; **9**(9): 1107-16.

18. Tong A, Sainsbury P, Craig J. Consolidated criteria for reporting qualitative research (COREQ): a 32-item checklist for interviews and focus groups. *International J Qual Health Care* 2007; **19**.

19. Chikovore J, Pai M, Horton KC, et al. Missing men with tuberculosis: the need to address structural influences and implement targeted and multidimensional interventions. *BMJ Global Health* 2020; **5**(5): e002255.

20. Majombozi Z. Care, contagion and the good mother: narratives of motherhood, tuberculosis and healing. *Anthropology Southern Africa* 2019; **42**(4): 290-301.

21. Daftary A, Calzavara L, Padayatchi N. The contrasting cultures of HIV and tuberculosis care. *Aids* 2015; **29**(1): 1-4.

22. Law S, Daftary A, O'Donnell M, Padayatchi N, Calzavara L, Menzies D. Interventions to improve retention-in-care and treatment adherence among patients with drug-resistant tuberculosis: a systematic review. *Eur Respir J* 2019; **53**(1).

23. Snyman L, Venables E, Trivino Duran L, et al. 'I didn't know so many people cared about me': support for patients who interrupt drug-resistant TB treatment. *Int J Tuberc Lung Dis* 2018; **22**(9): 1023-30.

24. Lönnroth K, Glaziou P, Weil D, Floyd K, Uplekar M, Raviglione M. Beyond UHC: Monitoring Health and Social Protection Coverage in the Context of Tuberculosis Care and Prevention. *PLOS Medicine* 2014; **11**(9): e1001693.

25. Nyblade L, Stockton MA, Giger K, et al. Stigma in health facilities: why it matters and how we can change it. *BMC Medicine* 2019; **17**(1): 25.

26. Daftary A. HIV and tuberculosis: the construction and management of double stigma. *Social science & medicine* 2012; **74**(10): 1512-9.

27. Wouters E, Sommerland N, Masquillier C, et al. Unpacking the dynamics of double stigma: how the HIV-TB co-epidemic alters TB stigma and its management among healthcare workers. *BMC Infectious Diseases* 2020; **20**(1): 106.

28. Link BG, Phelan JC. Stigma and its public health implications. *Lancet* 2006; **367**(9509): 528-9.

29. Szaflarski M. Spirituality and religion among HIV-infected individuals. *Current HIV/AIDS reports* 2013; **10**(4): 324-32.

30. Glover RE, van Schalkwyk MC, Akl EA, et al. A framework for identifying and mitigating the equity harms of COVID-19 policy interventions. *J Clin Epidemiol* 2020: S0895-4356(20)30597-7.

31. Brust JC, Shah NS, Scott M, et al. Integrated, home-based treatment for MDR-TB and HIV in rural South Africa: an alternate model of care. *Int J Tuberc Lung Dis* 2012; **16**(8): 998-1004.

32. Loveday M, Wallengren K, Brust J, et al. Community-based care vs. centralised hospitalisation for MDR-TB patients, KwaZulu-Natal, South Africa. *Int J Tuberc Lung Dis* 2015; **19**(2): 163-71.

33. Sinha P, Shenoi SV, Friedland GH. Opportunities for community health workers to contribute to global efforts to end tuberculosis. *Global Public Health* 2020; **15**(3): 474-84.

34. González Fernández L, Casas EC, Singh S, et al. New opportunities in tuberculosis prevention: implications for people living with HIV. *Journal of the International AIDS Society* 2020; **23**(1): e25438.

35. Guest G, Namey E, Taylor J, Eley N, McKenna K. Comparing focus groups and individual interviews: findings from a randomized study. *International Journal of Social Research Methodology* 2017; **20**(6): 693-708.

36. Zelnick JR, Seepamore B, Daftary A, et al. Training social workers to enhance patient-centered care for drug-resistant TB-HIV in South Africa. *Public Health Action* 2018; **8**(1): 25-7.

Table 1: participant characteristics/ ababambiqhaza______________

	Ingqikithi yabahlanganyeli (N = 55)
Ubulili<br>Abesifazane<br>Owesilisa	 **30 (55%)**<br>**25 (45%)**
Ubudala, iminyaka *	**35 (20-62)**
Uhlobo lwesifo sofuba<br>Ukumelana nemithi eminingi (MDR)<br>Ngaphambi kokulwa kakhulu nemithi (preXDR)<br>Imelana kakhulu nemithi (XDR)	 **35 (64%)**<br>**11(20%)**<br><br>**9 (16%)**
Isifo sofuba esedlule	**16 (29%)**
Umugqa wesibili owedlule womjovo	**14 (25%)**
Isikhathi sokwelashwa *<br>Ukwelashwa ngezidambisigciwane (ART)<br><br>I-DRTB †	 Izinyanga ezingama-23 **(**Izinsuku ezingama-27 kuya eminyakeni eyi-10)<br>Izinyanga ezine (izinsuku eziyi-17 kuya kwezingu-14)
Isikhathi esisele ekwelashweni kwe-DRTB*	**10 months (**Izinsuku ezingu-13 kuya ezinyangeni ezingu-22**)**
Isimo sokungena esibhedlela<br>Iziguli ezilaliswa esibhedlela<br>Iziguli eziphuma ngaphandle	 **23 (42%)**<br>**32 (58%)**
Umlando wokungena ‡<br>Ambulatory kuphela<br>Ulaliswe esibhedlela futhi uykwazi ukuzihambela<br>Isikhathi sokulaliswa esibhedlela, izinsuku §	 **4 (7%)**<br>**51 (93%)**<br><br>**86 (16-291)**
Imininingwane i-n (%) ne-median (range). DRTB = Isifo sofuba esingazweli emthini. * Ngesikhathi sokubamba iqhaza kweqembu. Ukwelashwa kwamanje ngomuthi osuselwa ku-bedaquiline ye-DRTB. ‡ Kuyo yonke inkambo yokwelashwa kwe-DRTB. §N = 51.	

Table 2: Conceptual categories of disruption (+) and amelioration (–) and representative quotes reflecting participants' experiences and perceptions during DRTB-HIV treatment /

Ithebula 2: Izigaba zomqondo zokuphazamiseka (+) Kanye nokuthuthuka (-) nezingcaphuno ezibonisa okwenziwayo nemibono yezigungu ngesikhathi sokwelashwa kwe-DRTB-HIV

	**Izingcaphuno**
**Isigaba 1: ukuxilongwa nokulaliswa esibhedlela — inkinga yokuqala**	
(-) Kumakwe njengokuhlukile; (-) ukucwaswa (uhlelo lwezempilo).	"Baphatha ngokwehlukile iziguli ezine-MDR, lapho bezwa nje ukuthi ngine-MDR baqala ukufaka izifonyo zabo bangihlalisa eceleni. " FG10_F
(-) Ukwehluleka kokwelashwa (isifo sofuba); (-) ukulahlekelwa yithemba.	Bengingazi noma kufanele ngiyeke ukuthatha [ukwelashwa kwamanje] ngokuphelele noma ngizilengise… ngicabange kimi ukuthi emva kwaso sonke lesi sikhathi sokuhlupheka, konke lokho bekungelutho. " FG11_F
(-) Ukuhlukaniswa nomndeni; (-) ukungakhoni ukuzikhethela noma ukuthola usizo.	"Ungitshele ukuthi angisazukubuyela ekhaya ngoba lokhu engikutholile kuyathathelana, ngathi, ngabe lokhu kusho ukuthi ngizohamba ngingasavalelisanga emndenini wami? Futhi uthe kumele ngikhethe ukufa noma lokhu. " FG10_F
(-) Ukucwaswa; (-) ukunganaki izibopho	"Bengizifihla kubasebenzi basemtholampilo… Ngesikhathi befika okokugcina bangithola bafika namaphoyisa… ngacabanga ukubaleka… [kodwa] kwakuphuthuma… ngaqiniseka ukuthi nginokuthile engingakwenzela [umndeni wami] uqhubeke ngaphambi kokuba ngihambe. " FG1_F
(-) Ukucwaswa (ekhaya)	"Ngacwaswa emndenini wami. Umuzwa wokungathandwa umndeni wakho… sengathi uyisilwane… Babengafuni ngihlale nabo… nokuchitha isikhathi nabo noma ukudla nabo. " FG1_F
(-) Ukucwaswa (umphakathi)	"Kube nokuthula lokhu okungazelelwe futhi akekho noyedwa oza ekhaya lami… Abantu bokuqala obekumele bangisekele bangishiyile futhi namanje ngingedwa. " FG6_F
(-) Ukucabanga nge-HIV	"Uma ubatshela ukuthi une-MDR bafika nesiphetho sokuthi nawe une-AIDS." FG1_F
(-) Alukho ulwazi (i-DRTB); (+) ulwazi (i-	"Sonke lesi sikhathi bengizizwa ngikhungathekile ngingazi ukuthi yini i-MDR… Kufanele bachaze ukuthi lezi yizinyathelo 1, 2, 3 no-4 okufanele

HIV); (+) imininingwane (ulimi)	uzilindele… Uma bengakwazi ukusinikeza ukwelulekwa njenge-HIV, ngizokwazi ukuqonda kangcono engikutholile, ngikuchaze kangcono emndenini wami futhi ngikhuthazeke ukuthatha imishanguzo. ” FG10_F; "Kuzoba ngcono ukuthi kube nomhumushi oqonda ulimi lwethu, ukuze sikwazi ukuzwakalisa izingqinamba zethu kangcono." FG3_M
(+) Ukunakekelwa ngozwelo; (+) ukuxhumana (abahlengikazi, ontanga); (+) kujwayeleke njengabanye	“Ekugcineni lapho ngifika lapha ngathola ukuthi ngisho nabahlengikazi bebenobungcono… Siyathanda lapho abahlengikazi behleka nathi ngoba kwesinye isikhathi sicabanga ukuthi bayikho ukusihlukanisa. Ugcina ungakhululekile kuleyo ndawo futhi lowo umuzwa obuhlungu kakhulu, ikakhulukazi lapho ubona ukuthi abantu abafuni ukuba nawe. Ngihlale izinsuku ezimbili lapha futhi ngacabanga, cha, lokhu kuhlangana kahle impela. ” FG1_F
**Isigaba 2: ukuqala ukwelashwa-ukuvalelwa**	
(-) Imithi emisha; (-) imiphumela emibi emisha (ngokomzimba noma ngokwengqondo); (-) amaphilisi amaningi (weDRTB, ne-HIV)	“Ibala [lami] lashintsha laba mnyama bhuqe… [ng] ahlanza kakhulu ... futhi ngaba nekhanda elibuhlungu ngenkathi ngelashwa i-MDR. Izinyanga ezimbalwa zokuqala, bona kunginike isikhathi esinzima kakhulu ... bekufanele ngilale usuku lonke… nami bengincipha kakhulu. Kwakukhona i-bedaquiline, ibibulala amalunga ami… ngicabanga ukuthi mina kumele ngilahlekelwe yingqondo lapho udokotela engitshela ukuthi kufanele bashintshe imishanguzo yami yegciwane lengculaza… bengeze amanye amaphilisi ayisihlanu [kuye kwayi-18 e-DRTB], manje mangaki lawo? ” FG11_F
(-) Ukuhlukana nengane; (-) ukulahlekelwa kwe-imali engenayo; (-) izibopho ezilahlekile	“Kuyethusa ukuzwa ukuthi ngeke usakwazi ukuqabula ingane yakho ikakhulukazi uma uyikhumbula, futhi ingane izothini lapho unina engakwazi ukuqabula yena? ” FG1_F; “Bengiwumshayeli kodwa ngaphelelwa umsebenzi… Umnikazi waqasha omunye umuntu ngenkathi ngigula ngakho ukuba lapha isikhathi eside kuzoholela ezinganeni zami ukuhlupheka ngoba ngiyabondla. ” FG3_M
(-) Ukunqanyulwa komndeni; (-) ukwesaba ukufa	“Uzizwa ungenamsebenzi njengoba ungaboni ngisho nomndeni wakho ... Ukubona abantu befa phambi kwakho kuyakwesabisa kodwa ukube ubusekhaya ubungeke ukubone lokhu. Ubuzogxila ekuphuzeni imithi yakho futhi uphile. ” FG1_F
(+) Imininingwane (HIV); (-) ukuntuleka kolwazi (nge-DRTB)	"Emnyangweni we-HIV sitshelwa ngomthamo wegciwane egazini kanye nesibalo se-CD4 ... Ukutshelwa ngentuthuko yakho kumkhuthaza kakhulu umuntu ... noma yini ngemiphumela yami yesikhwehlela, nemiphumela yami ye-MDR. ” FG2_F; "[Abahlinzeki]

	bathe akumele sibabuze… 'Anazi lutho'. Bathe ingxenye yayo yemithi ye-TB. ‘Ungabuzi imibuzo’. ” FG5_M
(-) asikho isikhuthazo noma ithemba<br>(-) Ukucwaswa (uhlelo lwezempilo)	"Awutholi ngisho umuntu oyedwa ukuthi akweluleke futhi akushukumise ukuthi yini okudingeka ube ngcono ukuze ungazitholi ufana ngesimo, nokukunikeza ithemba. ” FG1_F<br>“Uma ugula kakhulu, [abahlinzeki] bayahleba ngawe bethi wehlulekile ukwelashwa. Ukubizwa ngokuthi uyisephutha kubuhlungu ngempela ngoba bayabona uphuza imithi nsuku zonke. ” FG8_F
(-) Ukwamukelwa isikhathi eside; (+) ukunakekelwa ngozwelo; (+) isikhuthazo; (+) uxhumano (abahlengikazi, ontanga)	"Bakutshela ukuthi akumele uphumele ngaphandle kwewadi, futhi uzizwa njengesiboshwa." FG2_F; “Ngangilapha cishe izinyanga ezine ... okwaphula inhliziyo yami kodwa kwakukhona udade ongumhlengikazi owayengiphatha ngokungathi ngiyingane yakhe… Nguye kuphela umuntu owayenginaka futhi wayenginika ithemba futhi angitshele nami ukuthi nalokhu kuzodlula. ” FG6_F; "Ukukhuluma nabanye abantu kukusiza uzizwe ungcono, ikakhulukazi ukukhuluma nabantu abasesimweni esifanayo nesakho." FG1_F
(+) Umthandazo, ukholo	“Noma nini lapho ngangithatha [imithi], ngangithandaza kuqala futhi ngithole ukuthi lokho kwangisiza. Ngangithi, ‘Nkulunkulu, uyazi ukuthi kungani ungibeke kulesi simo futhi uyazi ukuthi uzongikhipha kanjani ’.” FG11_F
(+) Ukuvikelwa komndeni	“Ngilapha ngoba ngiwuthanda kakhulu umndeni wami, bengingafuni ukuthi bagcine begula futhi babe nalesi sifo… Ngifuna nje ukuthi lesi sifo sigcine ngami njengoba kuqale ngami. ” FG4_F
(+) Ukwesekwa kwezibhedlela; (+) ukunamathela emithini	"Ukungeniswa esibhedlela kungisize kakhulu, manje sengiyazi ukuthi ngo-8 ekuseni kumele ngiphuze umuthi wami." FG5_M; “Inhliziyo yakho iyakutshela ukuthi kufanele ube sekhaya, kodwa ekujuleni kwenhliziyo uyazi ukuthi ngeke ukwazi ukuthobela imithetho uwedwa. ” FG2_F
	(Table 2 continues on next page / Ithebula 2 liyaqhubeka ekhasini elilandelayo)

	**Izingcaphuno ezimele**
(Continued from previous page / Kuqhutshwa ekhasini langaphambilini)	
**Isigaba 3: ukukhishwa esibhedlela - ukuphumula nokuphazamiseka okuvuselelayo**	
(+) Ukunciphisa imiphumela engemihle; (-) kuyaqhubeka okuqhamuka uma udla imishanguzo; (-) amaphilisi amaningi (we-DRTB)	"Ekuqaleni kokwelashwa kunemiphumela engemihle oyitholayo. Njengoba uqhubeka, iyancipha… Ngisho namanje ngithola amava athile, kepha bona azinzima kangako. " FG2_F; "Ngangijwayele ukuthi umhlengikazi anginike umuthi, ekhaya kwakufana nokuthi imithi yami miningi kakhulu! Ngaqala ukucasuka futhi ngathukuthela lapho ngiphuza khona imishanguzo yami… Bekuzwakala sengathi imiphumela emibi ebengikade ngiyizwa ibuya njengesiyaluyaca… maningi kakhulu. " FG2_F.
(-) Ukululama kancane; (-) ukukhathala isikhathi eside; (-) ukulahleka kosizo; (-) intukuthelo yomndeni; (-) ukulahlekelwa ukwesekwa ngumndeni	"Ekuqaleni umama wakhombisa umdlandla ngokungisiza ngiphuze amaphilisi kodwa uyasebenza futhi akakwazi ukuhlala ekhona eduze nami … imiphumela emibi miningi futhi ... ngeke ngivele ngibonakale njengongenzi lutho ngakho manje kunzima ngoba angiphumuli ngokwanele. " FG10_F; ["Abantu] banenkolelo yokuthi uma usufikile emuva esibhedlela kufanele welapheke ngokuphelele. Bayakhohlwa ukuthi ukwelapha lesi sifo inqubo ende… Bakuphatha kahle ekhaya ezinyangeni zokuqala kodwa ngemuva kwezinyanga ezimbalwa izinto ziyashintsha. Bazosho izinto ezinjengokuthi 'udla njalo, uyavilapha', bakhohlwe ukuthi lesi yisifo esithinta izinyawo zakho sizenze zibe buhlungu futhi kuthatha isikhathi ukuthola amandla akho. " FG5_M
(-) Ukulahlekelwa isimo emphakathini; (-) ukulahlekelwa abangani	"Kubuhlungu lapho abangani bami bengishayela ucingo bethi asambe siye kule ndawo futhi kumele ngibatshele ukuthi angikwazi ukuza, kufanele ngihlale ekhaya. Ngicabanga ukuthi lokhu kuyalimaza kakhulu ngalesi sifo. " FG11_F
(-) Ukuphela kwenhlonipho, ukubaluleka; (-) ukulahlekelwa yisikhundla njengomlisa; Ukucwaswa (ekhaya, emphakathini)	"Nginomshana wami ohlale ephuma egumbini noma nini lapho ngikhona… Angikwazi nje ukubekezelela ukungahlonishwa yingane yentombazane… uyazi ukuthi anginamali. Lokhu kuziphatha kungenza ngizizwe sengathi angiyona indoda. " FG5_M; "Lapho kufika izingane zikamakhelwane wami, zibuye zibizwe," Uyazi ukuthi owesifazane wakuleyo ndlu uyagula! 'Konke lokho akungihlali kahle." FG10_F
(-) Ukulahlekelwa ukwesekwa esibhedlela	"Uxhaso oluthola esibhedlela alufani noluthola ekhaya. Esibhedlela, ukudla kuza ngesikhathi. Uma kungu-8 bese wena kuthole ngo-8. Njengoba ekhaya basengapheka ngo-8… futhi ngaleso sikhathi awukadli kusukela ekuseni. " FG7_M "Into uma usesibhedlela uwukhumbuli kakhulu umndeni wakho, ukhumbula impilo yakho

	ekhaya… kodwa futhi uyakhumbula ukuthi uzophathwa kabi lapho ubuya. ” FG5_M
(+) Ukuxhumana nomndeni; (+) umndeni ukwesekwa; (+) ukusekelwa kokubambelela	“Kuhle kakhulu ukubuyela ekhaya… Omalume bami bathenga ukudla okwanele ngathi futhi ngemali encane engiyitholayo ngiyazithengela izinto ezinhle engijabulela ukuzidla… Amaphilisi ami ahlala etafuleni lekhofi. Ngisho nomalume bami bazohlekisa ngakho bese bethi, ‘Lawa amaswidi akho mshana wami, sifisa sengathi nathi singabathatha’. ” FG6_F
(-) Ukutholakala kwezempilo; (-) ukuya emtholampilo; (-) ukumakwa njengohlukile	“Ngijwayele ukuya emtholampilo wangakithi… mhlawumbe umkhuhlane noma izinyawo ezibuhlungu… Bazokuphatha ngendlela ehlukile… bangitshele ukuthi abazi ngalesi sifo. Yebo, kepha mina angizile lapha ukuthola ikhambi lalesi sifo! … Ugcina ngokuxabana bese uphuma emtholampilo ungalutholanga usizo. ” FG7_M; “Ukufika lapha [esibhedlela sezifo ezijulile] kudinga ukuthi ngichithe ubusuku esibhedlela sakhona bese ngithuthwa yi-ambulensi ekuseni ukuyolanda umuthi… ngichitha izinsuku ezimbili ukuya nokubuya. Silala emabhentshini… omunye uzogcina elele phansi. ” FG3_F
(-) Alukho ulwazi; (+) ukukhipha ukwelulekwa	“Uma udokotela azi ukuthi usuzodedelwa… abahlengikazi noma abasebenzi lapha basifundise ngemithi yethu… bangakwenza ujwayeze… ukuze ungabi nezinkinga uma ubuyela ekhaya… ngoba abantu abaningi badidekile. ” FG12_M
(+) Imininingwane (yesiguli, umndeni, umphakathi)	“Sidinga ukuba nemikhankaso eminingi njengoba benza ngengculazi, abantu abasuka ezindaweni ngezindawo bafundise abantu ngalesi sifo… futhi kufanele abantu abake baba ne-TB phambilini ukuze sabelane ngokwenzeka, yiqonde ngqo kubantu. ” FG7_M
**Isigaba 4: ukuqhubeka nokwelashwa-akunakubonwa**	
(+) Ukubambelela okujwayelekile	“Ukuthatha umuthi wami kujwayelekile kimi manje, kusegazini lami. Kufana nokugeza, akekho umuntu okumele akutshele lokho. Ngiyakwazi okufanele ngikwenze. ” FG5_M
(-) Imiphumela emibi eqhubekayo; (-) kuyabonakala okuqhamuka uma udla imishanguzo	“Lobu bunzima bokubona into busangikhathaza… angikwazi ukufunda okuthile okubhalwe phansi. Bese kuba izinyawo zami. Kuzwakala sengathi kunefindo ngemuva kungivimbela ukuba ngihambe ngokukhululeka nangokushesha. ” FG9_M; "Uma ngihlangana nabantu bayabuza," Yini okungahambi kahle ngamehlo akho. Abomvu. Udakiwe? ... Wena udinga ukubuyela esibhedlela. Akufanele ube nathi lapha ngaphandle. ’… Umama wezingane zami wathi uma nje ngisahleli kuleli philisi, kufanele ngizitholele enye indawo. ”

	FG9_M; "Wonke umuntu uyangibuza uthi," Kwenzekeni kuwe? Kungani usube mnyama ngebala? ’Futhi kakhulu okucasulayo ukubachazela ukuthi ngidla amaphilisi. ” FG6_F
(-) Ukulahlekelwa yisibonelelo sikahulumeni; (-) ukuncika kwabanye	“Ngidonsa kanzima ngokwezezimali futhi kwesinye isikhathi ngiyafisa ukubuyiselwa emsebenzi wami. Udokotela wenqaba ngokuphelele ukusidlulisela izibonelelo, amaphilisi angenza ngidle kakhulu futhi ngiba umthwalo kulabo abahola imali ekhaya. ” FG6_F; “Ugcina usungumthwalo… kunzima ukuboleka imali kubantu ungasebenzi. Ugcina wenza izikweletu eziningi ukukhokhela esinye isikweletu. ” FG5_M
(+) Ukuqashwa; (+) ukuhamba; (-) ukuphazamiseka ekuphuzeni imithi	“Isimo singaba ngcono uma sinikezwa umsebenzi wesikhashana ukuze sikwazi ukufaka isandla ekhaya… Uma unenhlanhla yokuthola isibonelelo sokukhubazeka ositholayo ukuthi sihlal kuphela izinyanga eziyisithupha kanti ukwelashwa kwayo kuyiminyaka emibili. ” FG5_M; “Ngaqala ukusebenza [edolobheni elisha] ngakho ngangingajwayele imitholampilo yabo ... isikhathi esimatasa, kwesinye isikhathi umsebenzi wami ubuphela ngo-12 ebusuku ngakho angilitholanga ithuba lokulanda imuthi yami. ”FG5_M
(-) Ukuthola ukunakekelwa; (-) ukucwaswa (ezempilo uhlelo)	“Kungaba ngcono ukufinyelela amaphilisi ethu njengeziguli ezithola ama-ARV ezitolo nasemitholampilo yendawo… ungacela ngisho nengane ukuthi iyokulandela wona. ” FG7_M; “Ukuqoqa [ukwelashwa] lapha kungcono… Ukube bekungulayini walabo abane-MDR emtholampilo wendawo, ungahlala ucwaswa. ” FG10_F
(-) (+) Ukwesaba ukufa; (+) ukugxila ekululameni; (+) ukwenqaba ukucwaswa	"Yinto ecasula kakhulu ikakhulukazi ukuthatha leyo ye-TB, siyaziphoqa ukuthi siyiphuze ... Ukube angibonanga umuntu owehluleka ukwelashwa nokufa phambi kwami, nami ngabe ngehluleka ... kodwa ngiye ngabamukela futhi ngazitshela ukuthi le yimpilo yami, kumele ngiyinakekele. ” FG6_F; "Okubalulekile kimi ukunamathela emithini yami futhi nokungakhathaleli imibono yabanye abantu ... Kumele wamukele nje ukuthi uyagula futhi unamathela umuthi wakho ukuphela kwento ezokusiza… ukugxila empilweni yakho. ” FG5_M
(+) Ithemba, isifiso sokuvelela	“Ngifisa ukusinda ukuze ngikwazi ukubuyela esikoleni ngoba sengiqalile ukufaka izicelo, ngifuna ukuqhubekisela phambili imfundo yami futhi ngizibone ngifeza izinhloso zami, ngifisa sengathi ngingathola lelo thuba lapho ngizibona ngigqoke imibala yami yokuthweswa iziqu. ” FG10_F

Ukurekhodwa kanye namanothi emihlangano yezigungu kubhalwe phansi ngazwi linye ngabaqoqi bedatha, baziwa, bahlolwa ngokunemba kungakapheli isonto leseshini ngayinye, futhi bahunyushelwa esiNgisini kusuka esiZulwini ngaphakathi kwamaviki amane. (-) Kusho umqondo ophazamisayo futhi (+) kuchaza umqondo wokulolonga. Ama-ARV = izidambisigciwane. DRTB = Isifo sofuba esingazweli emthini. F = owesifazane. FG = isigungu/iqembu lokugxila. M = owesilisa. MDR = ukumelana nemithi eminingi. I-TB = isifo sofuba.

Figure 1: Focus group topic guide/ Umdwebo 2: Umhlahlandlela wesihloko seqembu lokugxila

Focus group main topics/ ezihlokweni eziyinhloko zeqembu

- Imibono ejwayelekile nge-DRTB, i-HIV, kanye nokutheleleka ngokuhlanganyela
- Imibono ejwayelekile ngempilo
- Ukuxilongwa kwe-DRTB ne-HIV (inqubo nokuphendula komuntu siqu)
- Ukudalulwa kokugula (yini, kubani, nini, nezimpendulo ezitholakele
- Okuhlangenwe nakho kuze kube manje ngokwelashwa (okuthandiwe, ukungathandwanga, izinkinga, nezinguquko ngokuhamba kwesikhathi)
- Ukusebenzisana namanethiwekhi omphakathi (izimpendulo nezinguquko ebudlelwaneni)
- Okuhlangenwe nakho kwezomnyango wezempilo (ukufinyelela ukunakekelwa, iziguli ezilala esibhedlela neziguli ezingaphandle, ukusebenzisana nabahlinzeki, uma kuqhathaniswa nokunakekelwa kwangaphambilini)
- Imibono ngezidingo nokwesekwa
- ukunakekelwa okulindelwe, okuthandwayo, nezikhala ekunakekelweni
- Olunye ushintsho empilweni yansuku zonke, indlela yokuzibuka, nezindlela zokuphila

Imibuzo ethuthukisa izingxoxo, nokuzibandakanya kweqembu (isb. ukuhlola okuqhubekayo ukuthola izincazelo ezigcwele (isib. ukucela ukwenaba, izibonelo, ukwehluka kwemibono)

Additional focus for groups in early-stage care

- Ukwamukelwa, ukungeniswa esibhedlela, kanye nolwazi lokuqalisa ukwelashwa
- Ubudlelwano bezenhlalo ngaphambi nangesikhathi sokulaliswa esibhedlela
- Izikhala kanye nokukhululeka ngesikhathi sokuqala kokuxilongwa nokwelashwa, nokudlulela esibhedlela

Additional focus for groups in mid stage care

- Ukukhishwa esibhedlela; ukubuyela ekhaya, emphakathini, noma emsebenzini; okuhlangenwe nakho kokuqhubeka kokwelashwa
- Ubudlelwano ngaphambi nangemva kokulaliswa esibhedlela, ukwelashwa kusenesikhathi naphakathi nendawo yesikhathi sokwelashwa
- Izikhala nokukhululeka ngesikhathi kuqhubeka ukwelashwa, nokubuyela ekhaya

Additional focus for groups in late stage care

- Ekhaya, umphakathi, emsebenzini; okuhlanganyelwe, nokuqhubeka kokwelashwa
- Ubudlelwano bezenhlalo ngemuva kokulaliswa esibhedlela, maphakathi nokwelashwa sekwedlule isikhathi
- Izikhala nokukhululeka ngesikhathi sokuqhubeka kokwelashwa, ekhaya, emphakathini, noma emsebenzini, nasemuva kokwelashwa

Figure 2. Chronology of disruptions during DRTB treatment in people with DRTB and HIV, and recommendations for amelioration/ Umdwebo 2: Ukulandelana kwezikhathi zokuphazamiseka ekwelashweni kwe-DRTB kubantu abane-DRTB ne-HIV, nezincomo zokuncishiswa

DRTB = Isifo sofuba esingazweli emthini.

Stage 1: diagnosis and hospitalisation—the first crisis/Isigaba 1: ukuxilongwa nokulaliswa esibhedlela — inkinga yokuqala

- Ukunakekelwa ngokuphuthumayo
- Ukwelashwa okubandlulululayo (izifonyo, ulayini)
- Ukulaliswa esibhedlela esikhulu
- Ukushiya ukuzibophezela emphakathini
- Ukwesaba okungaziwa, ukudalulwa kokugula
- Ukuntuleka kwemininingwane ne saziso, ukungabi nokuthetha

Stage 1: ameliorative strategies/Isigaba 1: Amasu wokuzikhuthaza

- Ukwelulekwa ngaphambi kokuhlolwa nangemuva kokuhlolwa
- Imininingwane ngezifo, ukwelashwa, imiphumela kanye nokuthelelekа
- Ukuqeqeshwa kwabahlinzeki bokunakekela okuyisisekelo
- Ukuzibandakanya komndeni

Stage 2: treatment initiation–displaced and confined /Isigaba 2: ukuqala ukwelashwa- ukuzizwa edukile nokuvalelwa

- Imiphumela engemihle, amaphilisi amaningi
- Ukuhlala esibhedlela isikhathi eside
- Ukunqanyulwa kumanethiwekhi womphakathi
- Ukungabi nandaba nomhlinzeki
- Ukwesaba ukufa, nokubona abantu befa
- Ukuntuleka kolwazi, ukunganyakazeki, ukungabi nokukhetha

Stage 2: ameliorative strategies / Isigaba 2: amasu wokuzikhuthaza

• Ulwazi ngemiphumela yokwelashwa, izibuyekezo, ukuthelelekа, ukungeniswa esibhedlela
• Ukuqapha nokuphathwa kwemiphumela engemihle
• Ukuhlaliswa esibhedlela, ukuxhuma kontanga (amaqembu), umndeni (ukuvakashelwa, izingcingo), ukunakekelwa ngamaqembu onke esibhedlela (umsebenzi wezenhlalakahle, ukwelulekwa ngengqondo)
• Ukuba matasa, ukukhonza noma ukukholwa
• Ukuqeqeshwa kwabahlinzeki abakhethekile
• Ukusekelwa komndeni

Stage 3: discharge home – reprieve and resurgent disruption/Isigaba 3: ukukhishwa esibhedlela-ukuphumula nokuphazamiseka okuvuselelayo

• Ukuzinakekela, amaphilisi amaningi, ukuphathwa kwamaphilisi
• Imiphumela engemihle, ukukhubazeka ngokomzimba nangokwengqondo
• Ukunakekelwa kwezempilo okuhlukanisiwe
• Izibopho zenhlalo, okulindelwe emndenini kungafinyelelwanga
• Ukungalondeki kwezezimali
• Ukucwaswa (umtholampilo, umphakathi, umndeni)

Stage 3: ameliorative strategies/Isigaba 3: amasu wokuzikhuthaza

• Ukukhishwa esibhedlela ngaphambi kwesikhathi
• Ukuqapha ukwelashwa okuhlukanisiwe
• Ukukhishwa esibhedlela nokweluleka
• Ukuzibandakanya komndeni
• Ukusekelwa komndeni
• Izinhlelo zokuqwashisa umphakathi

Stage 4: treatment continuity—no end in sight/Isigaba 4: ukuqhubeka nokwelashwa-akubonakali ukuphela

• Imiphumela emibi eqhubekayo, ebonakalayo
• Ukunakekelwa kwezempilo okuhlukanisiwe
• Izibopho zenhlalo, okulindelwe emndenini okungafinyelelwanga
• Izikweletu
• Ukucwaswa, ukungazethembi

Stage 4: ameliorative strategies/Isigaba 4: amasu wokuzikhuthaza

• Ukuqapha ukwelashwa okuhlukanisiwe
• Ukulethwa kwezinsiza okuhlukile, izinketho
• Ukuphathwa kwemiphumela emibi
• Ukuhlela nokwelulekwa ngemuva ukwelashwa
• Ukusekwa, ukuzakhela imali nemisebenzi
• Izinhlelo zokuqwashisa umphakathi nomqashi
